# Whole genome sequencing and annotation of *Aspergillus flavus* JAM-JKB-B HA-GG20

**DOI:** 10.1038/s41598-023-50986-5

**Published:** 2024-01-02

**Authors:** Achyut Ashokrao Bharose, Sunil Tulshiram Hajare, Dhawale Ramesh Narayanrao, H. G. Gajera, Hrushna Kany Prajapati, Suresh Chandra Singh, Vijay Upadhye

**Affiliations:** 1 Marathwada Agriculture University, Parbhani, M. S. 431401 India; 2 College of Natural and Computational Sciences, Dilla University, 419, Dilla, Ethiopia; 3 Department of Biotechnology, College of Agriculture, Junagadh Agricultural University, Junagadh, 362001 Gujarat India; 4 Shri Krishna Pathology and ELISA Laboratory, Sumerpur, India; 5 Bilva Laboratories Pvt Ltd, BHU Varanasi, Varanasi, Uttar Pradesh India; 6 Parul University, Vadodara, Gujarat India

**Keywords:** Biotechnology, Molecular biology, Plant sciences

## Abstract

Groundnuts are mostly contaminated with the mold *Aspergillus flavus* which produces a carcinogenic mycotoxin called as aflatoxin. It is very important to understand the genetic factors underlying its pathogenicity, regulation, and biosynthesis of secondary metabolites and animal toxicities, but it still lacks useful information due to certain gaps in the era of modern technology. Therefore, the present study was considered to determine the key genes and metabolites involved in the biosynthesis of aflatoxin by using a molecular approach in a virulent strain of *Aspergillus.* The whole genome sequence of highly toxic and virulent *Aspergillus* isolates JAM-JKB-B HA-GG20 revealed 3,73,54,834 bp genome size, 2, 26, 257 number of contigs with N50 value of 49,272 bp, 12,400 genes and 48.1% of GC contained respectively. The genome sequence was compared with other known aflatoxin producing and non-producing genome of *Aspergillus* spp. and 61 secondary metabolite (SM) gene clusters were annotated with the toxic strain JAM-JKB-BHA-GG20 which showed similarity with other *Aspergillus* spp. A total number of eight genes (ver-1, AflR, pksA, uvm8, omt1, nor-1, Vha and aflP) were identified related to biosynthesis of aflatoxin and ochratoxin. Also, 69 SSR with forward and reverse primers and 137 di and tri nucleotide motifs were identified in the nucleotide sequence region related to aflatoxin gene pathway. The genes and putative metabolites identified in this study are potentially involved in host invasion and pathogenicity. As such, the genomic information obtained in this study is helpful in understanding aflatoxin gene producing pathway in comparison to other *Aspergillus* spp. and predicted presence of other secondary metabolites clusters viz. Nrps, T1pks etc. genes associated with a biosynthesis of OTA mycotoxin.

## Introduction

The groundnut, or peanut (*Arachis hypogaea*), is a species in the legume or bean categorized under the family Fabaceae. The groundnut was probably first domesticated and cultivated in the valleys of Paraguay^1^. *Aspergillus* is a large genus composed of more than 180 accepted anamorphic species, with teleomorphs described in nine different genera^2^. The genus is subdivided in 7 sub genera, which in turn are further divided into sections^3^. Groundnuts are known to colonize and contaminated by *Aspergillus* spp. at pre and post harvesting stage^4^. Development of resistant varieties to seed invasion by *Aspergillus* spp. is one of the feasible means to minimize the aflatoxin contamination during groundnut storage with no extras input cost for the farmers. *A. flavus* is a fungus pathogenic to important agricultural crops and its products are a key concern on human health due to its ability to secret and synthesize the hazardous secondary metabolite called as aflatoxin^5,6^. The contaminated peanut with the mold *A. flavus* which produces a carcinogenic substance called aflatoxin B1 is considered to be the major cause of liver cancer and other chronic and acute diseases related to aflatoxin poisoning^7,8^. Stunting in children also reported due to sub lethal chronic exposure to aflatoxin^9^. *Aspergillus* genus is an important fungal genus with prolific sources of secondary metabolites of industrial and commercial importance^10^. *A. flavus* is well-known mycotoxin, ochratoxin A (OTA) producing species^11^. OTA is a mycotoxin possessing immunosuppressive and carcinogenic properties in humans as well as in animals and is responsible for oxidative DNA damage^12^. In rodents, OTA can generate life-threatening renal adenomas and hepatocellular carcinomas^13^.

It is very crucial to understand the regulation and biosynthesis of secondary metabolites due to their importance in plant pathogenesis and animal toxicities but still hindered by important information gaps. *A. flavus* possess 56 SM biosynthesis gene clusters^14^, but very few secondary metabolites have been assigned to a particular gene cluster. *A. flavus* hence could produce metabolites other than well-known mycotoxins that could be underrated contributors to its toxicity to humans and animals. It is a challenging task to develop aflatoxin resistant groundnut varieties due to the non-availability of reliable resistance sources, lack of information on plant-fungus interactions, and different environmental factors.

The whole genome of *A. flavus* was sequenced^15^, and annotation of the genome of the fungus showed various proteins, genes and other regulatory factors that are potentially related to conidial development and aflatoxin biosynthesis^16^. In the previous study, large numbers of strains were found non-aflatoxigenic while testing the aflatoxin-producing ability of *A. flavus*^17^. The ability of fungal species to produce aflatoxins is strain-specific and a different source of the strain that contains a different virulence genetic material^18,19^. Mostly such a variation may be due to hindrance in the aflatoxin biosynthesis genes or belong to other non non-aflatoxin producing species. The sequencing of aflatoxin biosynthesis gene cluster was extensively studied in order to understand the mechanism and biosynthesis pathway of aflatoxin in aflatoxigenic fungi^20,21^. The presence of these genes is required by the *Aspergillus* spp. to produce aflatoxin, and any changes therein might cause disruption in the biosynthetic pathway.

*A. flavus* is a major threat to food safety in study region with warm and humid climates that are affected by high levels of contamination. In this study, we sequence of highly toxic and virulent *Aspergillus* isolates JAM-JKB-B HA-GG20^22^ and also compared with other known aflatoxin producing and non-producing genome of *Aspergillus* spp. and total eight genes and 61 secondary metabolite (SM) gene clusters were annotated with toxic strain JAM-JKB-BHA-GG20 which showed similarity with other *Aspergillus* spp. Our analysis is useful to understand the aflatoxin gene pathway in comparison to other *Aspergillus* spp. and predicted the presence of other secondary metabolites clusters viz. Nrps, T1pks, etc. genes associated with biosynthesis of the OTA mycotoxin. The outcomes of the present study are briefly highlighted and thoroughly described below with important outputs.

## Results

### Screening of *Aspergillus* for aflatoxin using groundnut variety GG-20

Out of 21 *Aspergillus* isolates, 12 isolates were found to produce aflatoxin in the biochemical ammonium hydroxide test (Table 1; Additional file: Table S1).

**Table 1:** Total aflatoxin production by *Aspergillus* spp. (µg kg^−1^) after infections with GG-20 groundnut variety.

Time	5 days (µg kg^−1^)					10 days (µg kg^−1^)					15 days (µg kg^−1^)					Total (µg kg^−1^)
Isolate No. and code	B1	B2	G1	G2	Total	B1	B2	G1	G2	Total	B1	B2	G1	G2	Total	
1. JND-VAD-VAD-GG45	2726	71	39	0	2836	4827	344	177	104	5452	24,601	1171	533	105	26,410	34,698
2 RJD-UPA-KUN-G-2	17,080	472	58	0	14,159	12,878	714	469	98	17,610	71,277	3467	2049	291	77,084	108,853
3. JAM-JKB-BHA-GG20	18,583	557	378	0	19,519	90,935	1863	1449	233	94,480	26,958	1398	775	139	29,271	143,270
5. DWK-DWK-GG20 A	1106	0	0	0	1106	7937	258	154	0	8349	14,996	667	547	0	16,210	25,665
9. JND- YARD-1-GG20	73	0	31	109	213	156	45	0	0	201	123	0	29	0	151	565
12. RJD-UPA-KUN-TJ37A	12,324	347	250	0	12,921	34,965	1532	1171	218	37,885	26,839	1153	1163	126	29,281	80,087
13. RJD-DHO-PAR-GG37	49	38	0	0	88	140	0	0	0	140	9238	3541	2483	267	15,528	15,756
14. RJD-DHO-KAN-GG45	12,392	344	210	0	12,946	85,091	3337	2301	321	91,050	16,846	643	376	0	17,866	121,862
15. JND-MAN-LIM-GG20A	0	0	0	0	0	694	45	89	0	828	8457	4774	2706	522	16,460	17,288
16. JND-MAN-LIM-GG20B	0	0	0	0	0	0	0	0	0	0	199	0	0	0	199	199
18. JND-MEN-MEN-TJ45	345	47	30	0	423	10,896	406	297	0	11,599	77	0	35	0	112	12,134
21 JND- JAU –ISS-1	0	0	0	0	0	71	55	32	108	266	11,204	427	305	0	11,936	12,202
S.Em. +	95.82	1.12	0.87	0.16	1.85	2.87	1.88	1.68	1.34	1.44	2.54	2.43	1.85	1.67	1.11	
C.D. @ 5%	279.65	3.28	2.54	0.46	5.39	8.38	5.50	4.92	3.90	4.21	7.43	7.08	5.41	4.89	3.24	
C.V. %	3.10	1.25	1.82	3.02	0.06	0.02	0.46	0.57	2.57	0.03	0.01	0.29	0.35	2.42	0.01	

### Aflatoxin detection by LCMS-Q TOF

The LCMS-Q TOF assay revealed gradual increase in aflatoxins production and accumulation up to 10 days incubation period and then decrease at 15 days incubation in isolates number 3 (JAM-JKB-BHA-GG20), 12 (RJD-UPA-KUN-TJ37A), 14 (RJD-DHO-KAN-GG45) and 18 (JND-MEN-MEN-TJ45) While, isolate number 1 (JND-VAD-VAD-GG45), 2 (RJD-UPA-KUN-G-2) and 5 (DWK-DWK-GG20A) showed increase in aflatoxins production and accumulation was observed up to 15 days. On the other hand, isolate number 13 (RJD-DHO-PAR-GG37), 15 (JND-MAN-LIM-GG20A) and 21 (JND- JAU –ISS-1) illustrates the production and accumulation of aflatoxins only at 15 days (Table 1).

Among *Aspergillus* isolates, isolate number 3 (JAM-JKB-BHA-GG20) was found to be the most toxic, which produced high level of total aflatoxin (1, 43, 270 µg kg^−1^) followed by isolate 14 (RJD-DHO-KAN-GG45) (1, 21, 862 µg kg^−1^). The lowest level of aflatoxin was produced by isolate 9 (JND- YARD-1-GG20) and 16 (JND-MAN-LIM-GG20 B) (565 µg kg^−1^ and 199 µg kg^−1^, respectively). The comparative chromatogram represented the high production of B1 aflatoxin among isolates (Additional file: Figure S1A). Thus, the isolate 3 (JAM-JKB-BHA-GG20) was selected as a highly virulent and toxigenic aflatoxin producing *Aspergillus* which was collected from Village: Bhanvad; Taluka: Jamkhubhalia and District: Jamnagar from groundnut variety GG-20 (Table 1). Hence, isolate number 3 was used for further studies.

### Gene specific molecular characterization

For the differentiation of aflatoxin producers and non-producers, gene specific PCR was tested with several genes related to the aflatoxin biosynthetic pathway. The list of synthesized primers along with amplicon size is depicted in Table 2. A total 21 different isolates of *Aspergillus* were screened for aflatoxin production by using specific primers. Out of 21, 8 samples showed the aflatoxin production by amplifying the three target genes (Additional file: Figure S1B).

**Table 2:** Aflatoxin producing gene specific primers and their PCR products.

Primer	Gene	Primers sequences (5′—3′)	Total bases	PCR product size (bp)	
				Ref	Present findings
Tub1-F	tubl	5′ GCT TTC TGG CAA ACC ATC TC 3′	20	1498	1495
Tub1-R		5′ GGT CGT TCA TGT TGC TCT CA 3′	20		
Nor1-F	aflD	5′ ACG GAT CAC TTA GCC AGC AC 3′	20	990	985
Nor1-R		5′ CTA CCA GGG GAG TTG AGA TCC 3′	21		
OmtB(F)-F	aflO	5′ GCC TTG ACA TGG AAA CCA TC 3′	20	1333	1326
OmtB(F)-R		5′ CCA AGA TGG CCT GCT CTT TA 3′	20		
Omt1-F	aflP	5′ GCC TTG CAA ACA CAC TTT CA 3′	20	1490	1487
Omt1-R		5′ AGT TGT TGA ACG CCC CAG T 3′	19		

PCR was applied using four sets of primers (Table 5) for different genes involved in the aflatoxin biosynthetic pathway. Additional file: Figure S1B shows the PCR products obtained from each primer. Bands of the fragments of tub1, aflD, aflO and aflP genes can be visualized at 1495, 985, 1326 and 1487 bp, respectively. The tub1 gene producing band of 1495 bp was amplified in all 21 isolates (Additional file: Figure S1C). The housekeeping gene tub1 coding β-tubulin was served as control in the aflatoxin PCR study. The 3 genes aflD, aflO and aflP were tested as markers for discriminating between aflatoxin producers and non-producer. Out of 21 *Aspergillus* isolates, 08 isolates (isolate number 2, 3, 12, 13, 14 15, 16 and 21) showed similar pattern indicating the presence of the four genes and other strains failed to produce amplification patterns.

### Whole genome sequencing of identified most toxic *Aspergillus* isolate

The most toxic and highly virulent *Aspergillus* isolate JAM-JKB-BHA-GG20 was considered for the whole genome sequencing. The genome assembly is approximately 37.3 Mb in size with an N50 length of 49,272 bp (Table 3). Comparatively, *A. flavus* (JAM-JKB-BHA-GG20) had a similar GC% and proteins to *A. flavus* and the sister group *A. oryzae*, which differ considerably from the others.

**Table 3:** Contig measurements of genome of *A. flavus* JAM-JKB-BHA-GG20.

Particulars	Base-pairs
Total length	3,73,54,834
Count	6632
N75	25,320
N50	49,272
N25	84,433
GC %	48.1
Minimum	200
Maximum	2,26,257
Average	5633
Coverage	127X

### Quality coverage obtained from raw data of genome sequence

The number of sequences that support (cover) individual base positions is represented in Additional file: Figure S1D in that 100 percentages was observed at 100 bp positions and was good up to 300 bp. While, Additional file: Figure S1E indicates duplication level distribution, duplication levels are simply the count of how often a particular sequence has been found. Quality distribution observed at a base position above the median and percentiles of quality scores were neglected. The phred score median was found at 10^3^ indicating 1 ambiguous base out of 1000 bases (Additional file: Figure S1F).

### Trimming of whole genome sequence

Trimming of fungus genome sequences was done by CLC Genomics Workbench 9. The trimmed data showed 1, 172 bp (17.67%) trimmed reads with average trimmed length 30, 136. 3 bp (Table 4).

**Table 4:** Whole genome sequence trimming of *Aspergillus flavus* JAM-JKB-BHA-GG20.

Number of reads	6632
Average length	5632
No. of reads after trim	1172
Percentage trimmed	17.67%
Average length after trim	30,136.3

### Assembly of genome sequencing

**Figure 1:**
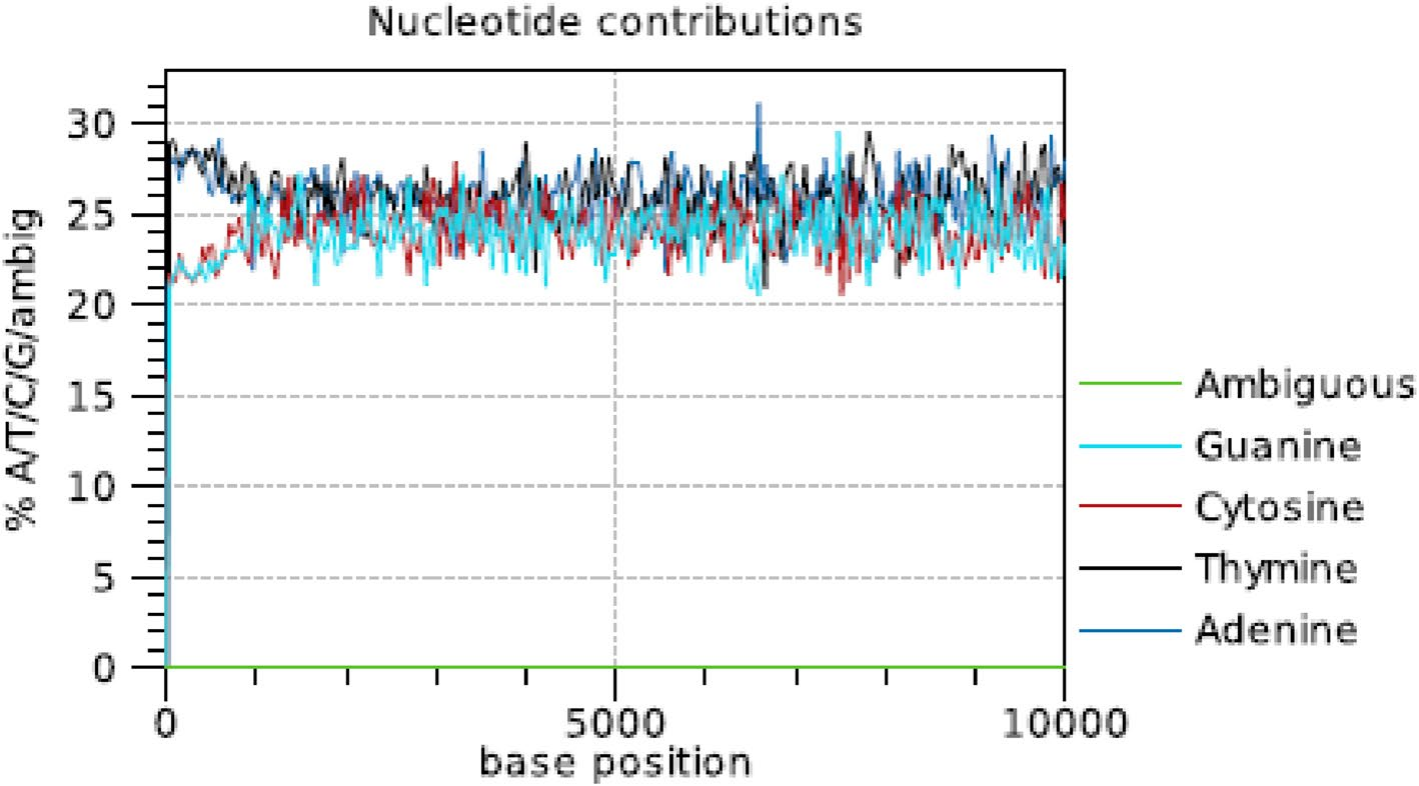
Coverage's for the four DNA nucleotides and ambiguous bases. x: base position; y: number of nucleotides observed per type normalized to the total number of nucleotides observed at that position.

After quality control of the raw NGS data, the most toxic and highly virulent *Aspergillus flavus* (JAM-JKB-BHA-GG20) genome assembly of reads was done by using CLC Genome Workbench 9. The assembled contigs smaller than 300 bp were discarded from the further analysis. The result of CLC Genome Workbench 9 showed maximum contig of 2, 26, 257 bp and minimum contig of 200 bp, with N75, N50 and N25 value of 25, 320 bp, 49, 272 bp and 84, 433 bp respectively (Table 5). N50 denotes a statistic that defines assembly quality. Given a set of contigs, each with its own length, the N50 length is defined as the shortest sequence length at 50% of the genome. The N75 and N25 statistic is less than or equal to the N50 statistic; it is the length for which the collection of all contigs of that length or longer contains at least 75% or 25% of the sum of the lengths of all contigs. The distribution of nucleotides in the raw sequence was 26.0% Adenine 24.0% Cytosine, 24.1% Guanine and 25.9% Thymine making 48.1% of GC (Fig. 1).

**Table 5:** Contig measurements of whole genome in *Aspergillus flavus* JAM-JKB-BHA-GG20.

Particulars	Base-pairs
Total length	3,73,54,834
Count	6632
N75	25,320
N50	49,272
N25	84,433
GC %	48.1
Minimum	200
Maximum	2,26,257
Average	5633
Coverage	127X

### Genome annotation of *Aspergillus flavus* JAM-JKB-BHA-GG20

The draft genome of the most toxic and highly virulent *Aspergillus* JAM-JKB-BHA-GG20 was annotated by using the widely used AUGUSTUS version 2.7. A total of 3, 73, 54, 834 bp genome was assembled, which consists of 2, 26, 257 contigs. The software annotated 12,400 genes from the total fungal genome with 12,717 mRNA (Table 6). Thus, while the number of genes might be slightly overestimated because of initio prediction limitations, at least 70.8% of the annotations were supported by experimental evidence. It had confirmed that the assembly was able to capture full-length genes by searching the predictions for full open reading frames (ORFs), finding that most of the genes contained start (12,500) and stop codons (12, 483) (Table 6). Sequence comparisons in RepeatMasker were performed by ABBlast/WUBlast search engine. The RepeatMasker search determined 7050 repeat regions in total genome of the most toxic fungus *A. flavus* (Table 6). Models of putative inter spread repeats were calculated by RepeatModeler which determined 416 repeat regions. Different putative clusters were found for this strain, suggesting that the *Aspergillus* fungus is capable of producing a great many more compounds than just the aflatoxins (Table 6). A total 5, 33, 371 open reading frame (ORF) were identified by using the program GETORF details, which is part of EMBOSS package. Further, 237 tRNA’s were sorted out by using tRNAscan software (Table 6).

**Table 6:** Genome annotation results of *A. flavus* JAM-JKB-BHA-GG20.

Program	Predicted features	Total
Augustus	Gene	12,400
	mRNA	12,717
	Start codon	12,500
	CDS	43,566
	Stop codon	12,483
	Intron	31,376
	Total	1,25,04
SSRs results	SSR	12,122
	Aflatoxin related SSR motif	137
	Aflatoxin related SSR primers	69
tRNA scan	tRNA	237
Getorf results	ORF	5,33,371
Repeat masker	Repeat region	7,050
Repeat modeler	Repeat region	416

### Gene identification related to aflatoxin biosynthesis pathway

Total 8 genes related to aflatoxin biosynthesis pathway were identified (Table 7). These include the *ver-1* gene, which is involved in the conversion of versicolorin A to sterigmatocystin, a regulatory gene, *aflR* (previously named *afl-2* for *A. flavus* and *apa-2* for *A. parasiticus*), that codes for a regulatory factor (AFLR protein) and involved in the activation of pathway gene transcription. The *pksA* gene which codes for a polyketide synthase whereas, a putative fatty acid synthase gene *uvm8* is potentially involved in polyketide backbone synthesis. The *omt1* gene coding for an *S*-adenosylmethionine-dependent *O*-methyltransferase that converts sterigmatocystin to *O*-methylsterigmatocystin and dihydrosterigmatocystin to dihydro-*O*-methylsterigmatocystin and the *nor-1* gene, which codes for a reductase that converts norsolorinic acid to averantin. *Vha and* aflP genes were potentially dehydrogenase involved in an intermediate step of aflatoxin biosynthesis. Genes were searched for the corresponding homologizes in NCBI data base. The BLAST results illustrated that the identified genes resembled close phylogenetic relationship to maximum (100%) similarity with *A. oryzae* and low (85%) similarity with *A. nidulans* etc. The genes annotation and their association with homologous nucleotide in different fungal classes are shown in Table 8.

**Table 7:** Identified gene related to aflatoxin biosynthesis pathway in *Aspergillus flavus* JAM-JKB-BHA-GG20.

Gene	Code no	Contig	Gene function	Location
*ver-1*	AF.00g044280	Contig_261	Involved in the conversion of versicolorin A to sterigmatocystin	99,814–101,526
*Afl R*	AF.00g031840	Contig_175	Codes for the regulatory factor (AFLR protein), controls the expression of all of the characterized structural genes (*nor-1*, *ver-1*, and *omtA*)	114,065–114,904
*pksA*	AF.00g013430	Contig_56	A gene, which codes for a polyketide synthase	37,144–38,788
*uvm8*	AF.00g022370	Contig_106	Potentially involved in polyketide backbone synthesis	43,309–44,455
*omt*1	AF.00g044990	Contig_238	Codes for an *S*-adenosylmethionine-dependent O-methyltransferase that converts sterigmatocystin to *O*-methylsterigmatocystin and dihydrosterigmatocystin to dihydro-*O*-methylsterigmatocystin	20,818–22,326
*nor-1*	AF.00g024610	Contig_137	Codes for a reductase that converts norsolorinic acid to averantin	19,243–20,544
*Vha*	AF.00g024610	Contig_137	Versiconal hemiacetal acetate (VHA)	19,243–20,544
*aflP*	AF.00g044990	Contig_238	Biosynthesis pathway gene	20,818–22,323

**Table 8:** Comparison of aflatoxin specific genes with other *Aspergillus* spp. based on blast identities and base pair score.

Genes	*ver-1*		*Afl R*		*pksA*		*uvm8*		*omt1*		*nor-1*		*Vha*		*aflP*	
	Blast identities (%)	Match pairs (bp)	Blast identities (%)	Match pairs (bp)	Blast identities (%)	Match pairs (bp)	Blast identities (%)	Match pairs (bp)	Blast identities (%)	Match pairs (bp)	Blast identities (%)	Match pairs (bp)	Blast identities (%)	Match pairs (bp)	Blast identities (%)	Match pairs (bp)
Test *A. flavus*	100	1713	100	840	100	1645	100	1147	100	1832	100	1302	100	1290	100	1509
*A. flavus* 3357	84.76	1452	99.64	837	86.57	1424	80.56	924	89.63	1642	37.21	995	98.37	1278	99.47	1501
*A. parasiticus* IC98	39.63	732	–	–	–	–	–	–	31.20	579	13.05	342	–	–	–	–
*A.oryzae* RIB 40	82.52	1449	89.07	839	79.42	1374	65.18	848	90.07	1650	98.77	1289	98.77	1291	48.75	1499
*A. nomius* NRRL 13,137	–	–	–	–	76.47	1258	–	–	80.94	1550	–	–	–	–	–	–
*A. niger* CBS 513	–	–	–	–	–	–	–	–	49.08	1222	–	–	–	–	–	–
*A. westerdijkiae*	–	–	–	–	–	–	–	–	–	–	–	–	–	–	–	–
*A. clavatus*	–	–	–	–	–	–	–	–	–	–	–	–	–	–	–	–
*A. fumigatus*	–	–	–	–	–	–	–	–	–	–	–	–	–	–	–	–
*A. terreus*	–	–	–	–	–	–	–	–	–	–	–	–	–	–	–	–
*N. fischeri*	–	–	–	–	–	–	–	–	–	–	–	–	–	–	–	–

### Comparative analysis of aflatoxin pathway genes

**Figure 2:**
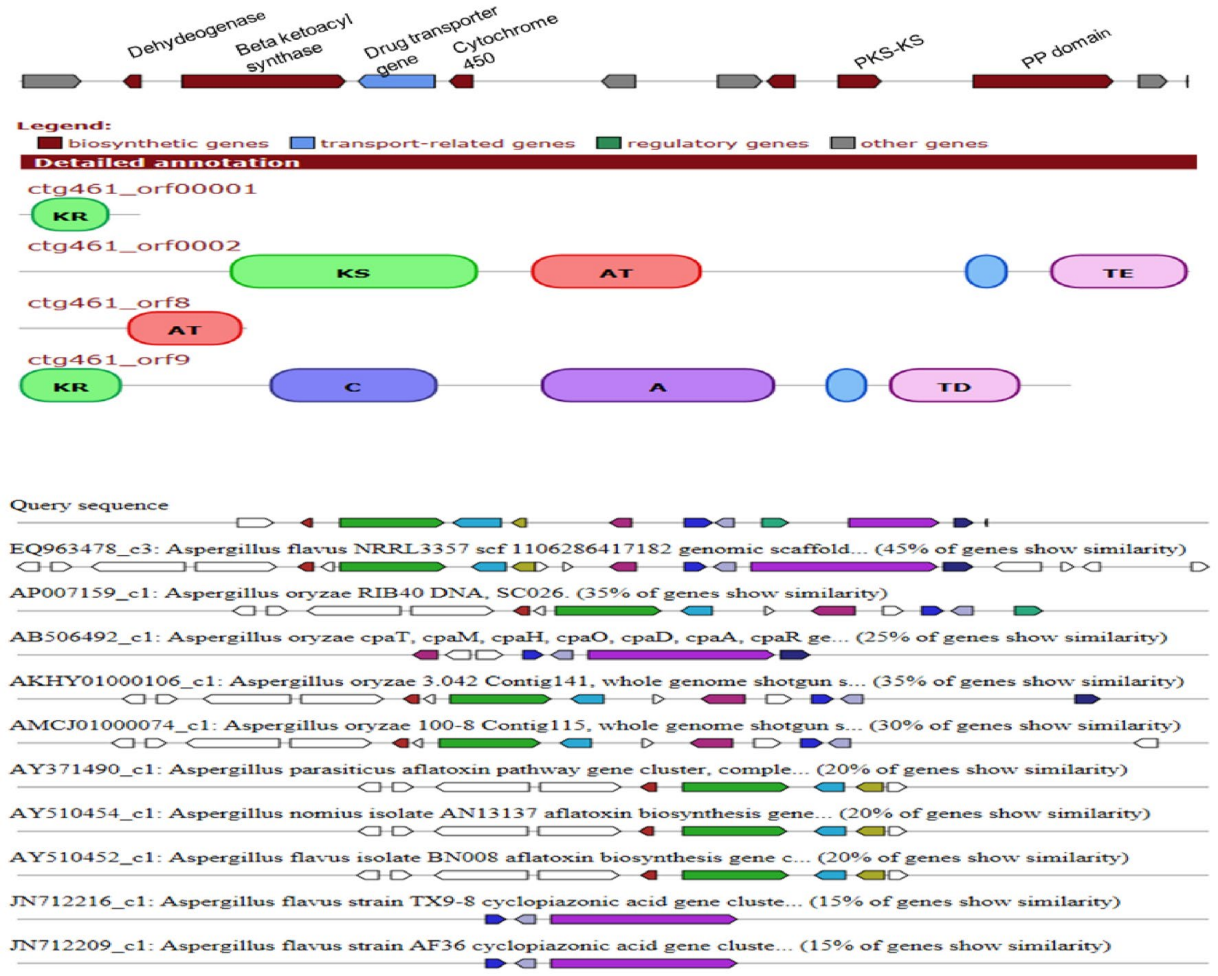
The putative cluster- 39 of *A. flavus* JAM-JKB-BHA-GG20 found in this study and its comparison aflatoxin biosynthesis gene of *A. parasiticus* (AY371490), *A. nomius* isolate AN13137 (AY510454) and *A. flavus* isolate BN008 (AY510452) isolates.

Pairwise comparisons between the aflatoxin pathway genes viz*. nor-1*, *pksA, ver-1* and *Uvm8* of *A. flavus* JAM-JKB-BHA-GG20 with *A. flavus* NRRL 3357, *A. parasiticus*, *A. nomius* NRRL 13,137 and *A. oryzae* RIB 40, was conducted by applying to the CLC Genomics Workbench 9 (Table 8; Fig. 2). The highest sequence similarity 99.64% was found with *A. flavus* NRRL 3357 whereas, the least sequence similarity 13.05% was found in *A. parasiticus nor-1*gene.

Dotplot comparison of the *ver-1* gene of *A. flavus* JAM-JKB-BHA-GG20 to *A. flavus* NRRL 3357 revealed sequence similarity (84.76%) and synteny over a region of at least 1452 bp, *A. parasiticus* revealed sequence similarity (39.63%) and synteny over a region of at least 732 (Additional file: Figure S1G).

**Figure 3:**
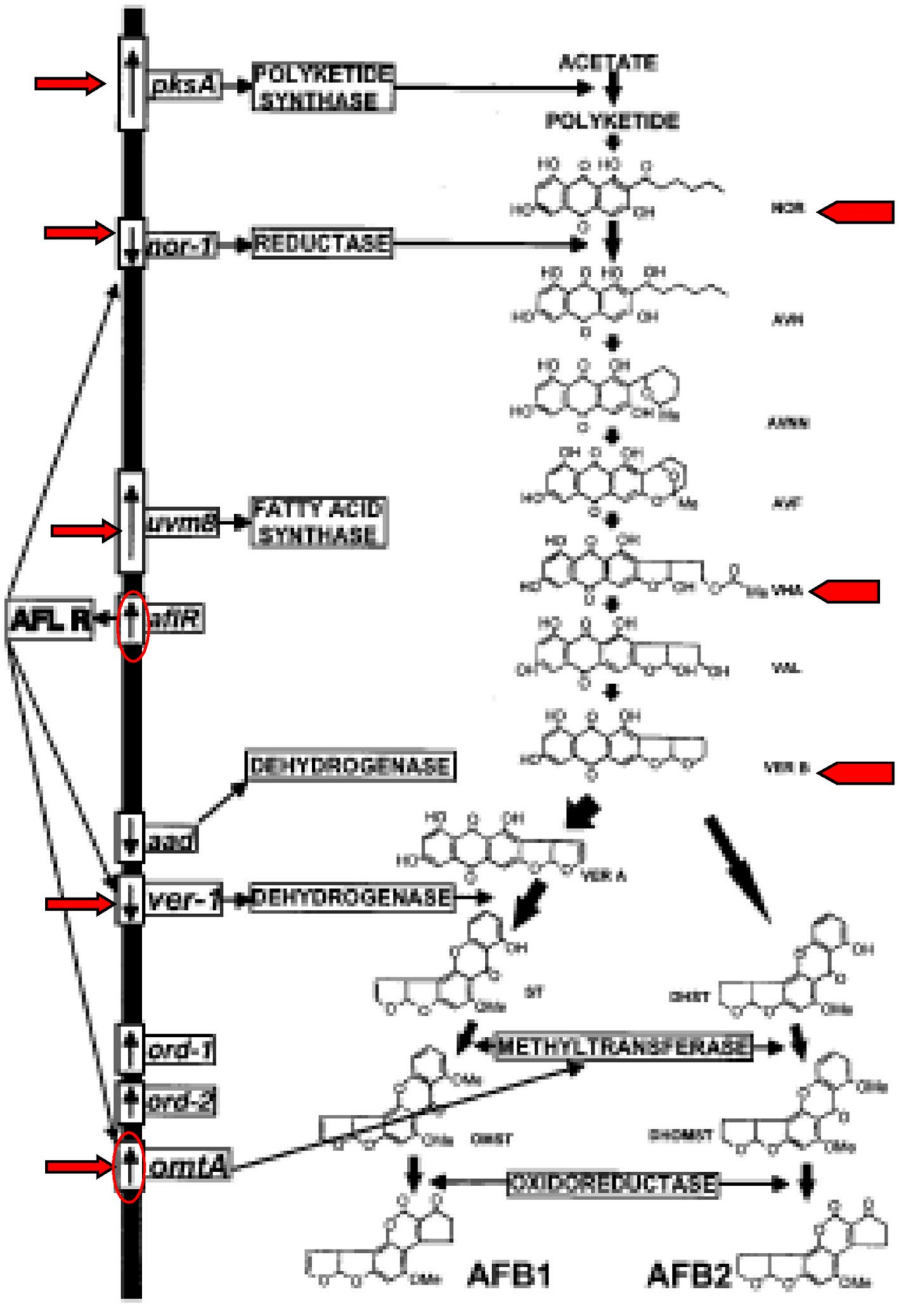
Genes and enzymes in the aflatoxin B1 and B2 biosynthetic pathway.

Dotplot comparison of the *pksA* gene of *A. flavus* JAM-JKB-BHA-GG20 to *A. flavus* NRRL 3357 revealed sequence similarity (86.57%) and synteny over a region of at least 1424 bp, *A. oryzae* RIB 40 revealed sequence similarity (79.42%) and synteny over a region of at least 1374 bp whereas, *A. nomius* NRRL 13,137 revealed sequence similarity (76.47%) and synteny over a region of at least 1258 bp (Additional file: Figure S1G). Dotplot comparison of the *Uvm8* gene of *A. flavus* JAM-JKB-BHA-GG20 to *A. flavus* NRRL 3357 revealed sequence similarity 80.56%) and synteny over a region of at least 924 bp, whereas, *A. oryzae* RIB 40 revealed sequence similarity (65.18%) and synteny over a region of at least 848 bp (Fig. 3). As such the nucleic acid similarity matches above 98% had a continuous straight diagonal line in the center of the matrix. Dotplot comparison of the *nor-1* gene of *A. flavus* JAM-JKB-BHA-GG20 to *A. flavus* NRRL 3357 revealed sequence similarity (37.21%) and synteny over a region of at least 995 bp, *A. parasiticus* revealed sequence similarity (13.05%) and synteny over a region of at least 342 bp whereas, *A. oryzae* RIB 40 revealed sequence similarity (98.77%) and synteny over a region of at least 1289 bp (Additional file: Figure S1G).

### Secondary biosynthetic gene clusters of *A. flavus* JAM-JKB-BHA-GG20

Using the whole genome of *A. flavus* JAM-JKB-BHA-GG20 as the query sequences of the antiSMASH 3.0 platform, many short and dense fragments are present in some SM gene clusters, suggesting low prediction accuracy. Therefore, SM gene clusters were constructed. Many of the putative SM gene clusters belong to type *Ipks* (*t1pks*) or non-ribosomal peptide synthase (*nrps*) gene clusters, while the remaining were terpene, indole, or other hybrid gene clusters (Table 9). The number of putative *nrps* gene (16) *pks* gene (14) and hybrid nrps/pks gene (07) of *A. flavus* JAM-JKB-BHA-GG20 were noted. The hybrid *T1pks-indole-nrps* gene cluster encoded Dehydrogenase, Beta ketoacyl synthase, Drug, cytochrome 450 genes. The genes present in this cluster showed 20% homology towards aflatoxin pathway gene clusters of *A. parasiticus* (AY371490), *A. nomius* isolate AN13137 (AY510454) and *A. flavus* isolate BN008 (AY510452) isolates whereas, 42% homology towards known gene cluster of *A. flavus* aflatoxin (BGC0000006, BGC0000008) AY510451 and AY510453 was acquired from Minimum Information about a Biosynthetic Gene cluster (MIBiG) database (Fig. 3). The generally accepted aflatoxin B1 and B2 biosynthetic pathway in *A. parasiticus* and *A. flavus*, enzymes for some specific conversion steps, and cloned genes are schematically presented. The regulatory gene, *aflR*, coding for the regulatory factor (AFLR protein), controls the expression of all of the characterized structural genes (*nor-1*, *ver-1*, and *omtA*). The *ver-1* gene product(s) has not been fully characterized; the catalytic steps in the aflatoxin biosynthetic pathway of the *uvm8* and the *aad* gene products, fatty acid synthase and a dehydrogenase, respectively, are not defined; and the *ord-1* and *ord-2* gene products are under investigation. The approximate sizes, relative locations, and directions of transcription of the identified genes, are indicated. Transcription of structural genes *nor-1*, *ver-1*, and *omtA* is regulated by AFLR. Abbreviations: NOR, norsolorinic acid; AVN, averantin; AVNN, averufanin; AVF, averufin; VHA, versiconal hemiacetal acetate; VHOH versiconal; Ver B, versicolorin B; Ver A, versicolorin A; ST, sterigmatocystin; DHST, dihydrosterigmatocystin; OMST, *O*-methylsterigmatocystin; DHOMST, dihydro-*O*-methylsterigmatocystin; AFB1 aflatoxin B1; AFB2, aflatoxin B2 (Fig. 3).

**Table 9:** Classification of putative secondary metabolites clusters of predicted by anti-SMASH.

Sr.No	Type	Count
1	Nrps	16
2	T1pks	14
3	Terpene	7
4	Indole	5
5	T3pks	3
6	Nrps-T1pks	2
7	T1pks-Indole-Nrps	1
8	Indole-Nrps	1
9	T1pks-Nrps	1
10	Siderophore	1
11	Other	10
	Total	61

## Discussion

Groundnut is grown on a large scale in almost all the tropicals and sub-tropical countries in the world. A groundnut is the third important oil seed crop of the world in production after the soybean and cotton^23^. India shares 23% of the world’s groundnut area and production. In India, it is grown over an area of 43.16 lakh hectares with total production of 51.27 lakh tonnes^24^. The groundnut crop is attacked by a number of diseases. The economically important foliar fungal diseases are early and late blight rusts etc. Seed and soil borne diseases like collar rot, stem rot and dry root rot are also restricted the production of groundnut. Viral diseases like bud necrosis, peanut mottle diseases etc. are also affecting groundnut producing^25^. Groundnut may be contaminated with the mold *Aspergillus flavus* which produces a carcinogenic substance called aflatoxin and it detonates the export quality of a groundnut. Aflatoxin accumulation and production by the fungus in groundnut poses a serious threat to human health and agriculture commodities. Variability in production of aflatoxins, especially among *A. flavus* isolates, has often been reported^25–28^. For example, only half of *A. flavus* strains produce aflatoxins, however, many of these strains produce more than 106 μg kg^−1^^29^.The production of aflatoxin involves a complex biosynthetic pathway consisting of at least 25 genes^30^. Identifying key genes and respective product involve in aflatoxin production has been a debate over the past issue. Therefore, the proposed study was aimed to identify key genes involve in highly infected *A. flavus* strain using molecular aspects in the selected area as the first approach.

Among 21 isolates studied, 12 of them were reported producing aflatoxin confirmed by biochemical test as well as LCMS-Q TOF. These strains are reported producing aflatoxin in the range of 1, 43, 270 µg kg^−1^ to 199 µg kg^−1^ till 15 days of infection. This report is somewhat similar or dissimilar to other studies presented earlier. The variation in the ability of isolates to produce aflatoxin may be due to genetic differences between fungal isolates as well as different physiological conditions^31^. Likewise, Harry and Hugo^32^ demonstrated the production or accumulation of aflatoxins in vitro by four isolates on three substrates (acid-delinted cottonseed, shelled Spanish peanut, and rough rice) with relation to temperature in the range of 100–400 °C. Within the first 10 days after inoculation, the optimal temperature range for aflatoxin production was between 200 and 350 °C. Only small amounts of the toxins were produced at 100 and 400 °C. Within the optimal temperature range, the time required for toxin production and for significant accumulation decreased as the temperature increased. More aflatoxin G was produced or accumulated in relation to aflatoxin B at low temperatures.

Nagarajan and Ramesh^33^ studied toxin production by the different fungal isolates varied considerably. Isolates of *A. flavus* produced only B1 and B2, whereas *A. parasiticus* produced B1, B2, G1 and G2. The results indicate clearly that there are species and varietal differences in toxin production. The variation in toxin production appears to be intimately related to the inherent ability of the fungal isolate to produce the toxin. The fungal isolates used by Rao and Tulpule^34^ was perhaps so low in toxin-producing capability that no detectable toxin could be detected when it was grown on US-26, although measurable amounts of the toxin were produced on other varieties of peanuts. The toxin production was also related to the species of *Aspergillus* used; *A. parasiticus* always produced greater amounts as compared to *A. flavus*.

Aflatoxins may be produced but not detected because of the inherent detection limits of the analytical systems. This approach aims at selecting a solid molecular marker for discriminating *Aspergillus flavus* species. For the differentiation of aflatoxin-producers and non-producers, gene specific PCR were tested with several genes related to the aflatoxin biosynthetic pathway. In this study, aflatoxin producing *Aspergillus* isolates were conformed for its toxicity with molecular approach. For diffracting isolated *Aspergillus* for aflatoxin producing and non-producing, the 3 genes viz. aflD, aflO and aflP specific primers were tested by earlier researcher^35^. The 3 genes aflD, aflO and aflP were tested as markers for discriminating between aflatoxin producers and non-producers^36^. Out of 21 *Aspergillus* isolates, 08 isolates such as isolate no. 2, 3, 12, 13, 14 15, 16 and 21 showed a similar pattern indicating the presence of the four genes and other strains failed to produce amplification patterns.

Researchers have reported use of PCR technology as rapid and sensitive method for detection and diagnosis of aflatoxin production, to detect aflatoxigenic strain from non-aflatoxigenic strain in other food commodities apart from the groundnut^37^. PCR analysis was able to amplify two structural genes i.e. nor1 and ver1 and regulatory gene aflR by using specially designed primers. The aflR is a positive regulatory gene which is required for transcriptional activation of most of the structural genes^38,39^.

Adeela et al.^37^ evaluated fungal flora of peanuts to test aflatoxin contamination. 10 isolates out of 40 were aflatoxin producers when tested by PCR analysis. The study revealed that nor1 gene was being amplified in almost all of them as it was a structural gene required in the initial step. Three primers were carefully selected to be highly specific for these three genes known to be essential for aflatoxin biosynthesis. Each primer pairs yielded a single DNA fragment of the expected size of 400, 600 and 1000 bp for nor1, ver1 and aflR respectively.

In present study, β-tubulin gene found to be expressed in all the isolates. Kanbe et al.^40^ reported single-copy conserved genes can also be used as targets for taxonomic studies within the *A. flavus* group, when multi-copy segments from the rRNA gene complex lack variability. Universal β-tubulin, calmodulin and topoisomerase II genes have been used in fungal species identification. Genes involved in secondary metabolism are considered to be more variable within closely related species^41^.

Yu et al.^36^ surveyed fourteen strains of *A. flavus* as a test sample and three samples of other fungi, such as *A. ngier, P. expansium, F. verticillioides*, which were assumed a positive control through TLC technique and PCR method, working with nor-1, ver-1, omt-1, aflR primers. Nor-1, ver-1, omt-1 are three structural genes in cluster genes in biosynthesis aflatoxin pathway that coding for key enzymes in production of aflatoxin, thus they are essential for aflatoxin production whereas, aflR is a positive regulatory gene required for activation of structural genes. The result revealed that three samples of fourteen strains of A. flavus were positive using TLC technique and totally twelve samples with the four mentioned primers used in PCR technique showed positive results. None of the other fungal strains using TLC and PCR did show any positive results. The positive control in both techniques was positive.

The similar results of tub1, aflD, aflO and aflP genes expression was studied which revealed those applied conditions were set up perfectly, as though each primer formed sharp and distinct bands in its specific area. Other studies suggest that regulation of aflatoxin biosynthesis in *Aspergillus* spp. involves a complex pattern of positive and negative acting transcriptional regulatory factors, which are affected by environmental and nutritional parameters^38,39^.

The same result has been achieved by using PCR and multiplex PCR procedures^42,43^ The interpretation of the results revealed that PCR is a rapid and sensitive method (sensitivity 100%, specificity 75%) in diagnosis of aflatoxinogenic molds but, this technique (PCR) cannot differentiate between toxic and nontoxic fungi. Geiser et al.^44^ suggests that the lack of aflatoxin production could also be due to simple mutations including substitution of some bases and Liu and Chu^45^ suggest that a variety of different physiological conditions affecting aflatoxin biosynthesis.

In previous study a multiplex real time-PCR protocol has been standardized based on aflD, aflO, and aflQ genes to differentiate aflatoxin-producing strains of *A. flavus* from the aflatoxin-nonproducing ones. They found a good correlation between the target genes expression in nearly all samples^38,39^.

The result of whole genome sequencing revealed approximately 37.3 Mb in size with an N50 length of 49,272 bp. Comparatively, the species isolated in our study had a similar GC% and proteins to that of *A. flavus* and the sister group *A. oryzae*, somewhat differ considerably from the others. A total of 3, 73, 54, 834 bp genome was assembled, which consists of 2, 26, 257 contigs. The software annotated 12,400 genes from total fungal genome with 12,717 mRNA. Thus, while the number of genes might be slightly overestimated because of initio prediction limitations, at least 70.8% of the annotations were supported by experimental evidences. It had confirmed that the assembly was able to capture full-length genes by searching the predictions for full open reading frames (ORFs), finding that most of the genes contained start (12,500) and stop codons (12, 483). Sequence comparisons in RepeatMasker were performed by ABBlast/WUBlast search engine. The RepeatMasker search determined 7050 repeat regions in total genome of most toxic fungus *A. flavus*. Models of putative interspread repeats were calculated by RepeatModeler which determined 416 repeat regions. Different putative clusters were found for this strain, suggesting that the *Aspergillus* fungus is capable of producing a great many more compounds than just the aflatoxins. The result are agreed with This agreed with the preceding research results of Nierman et al.^46^, who reported 5 × genome coverage, which was assembled into 958 contigs comprising 331 scaffolds ranging in size from 4.46 Mbp to 211 bp and containing a total of 958 contigs. The genome size 37 Mbp which matches with our findings *i.e.*37.3 Mb genome size. The average GC content was 44% which was less than our results (48.1%). The number of predicted protein-coding genes is 13,485. Similarly, Yoko et al.^47^ performed genome sequencing on a Pacific Biosciences RS II (Pacific Biosciences, Menlo Park, CA, USA) using libraries prepared with the SMRTbell template prep kit 1.0 (Pacific Biosciences). A draft genome of *A. lentulus* was assembled using SMRT Analysis 2.3 (Pacific Biosicences). Eventually, 19 scaffolds were obtained, summing up to 30,956,128 bp with an overall GC content of 49.45% greater than our report (48.1%). The *N*50 and maximum scaffold were 4,166,724 bp, and 5,062,644 bp, which are more as compare to our findings (49,272) respectively. Gene annotation using AUGUSTUS program 2.5.5 trained with the parameters of the species *Aspergillus fumigatus* resulted in 9860 genes. 200 tRNAs and 106 rRNAs were predicted by tRNAscan-SE 1.3.1 and RNAmmer 1.2, respectively. Maria et al.^48^ assembled a 54.9 Mb *P. lycopersici* draft genome sequence based on Illumina short reads, and annotated approximately 17,000 genes. The *P. lycopersici* genome is closely related to hemibiotrophs and necrotrophs, in agreement with the phenotypic characteristics of the fungus and its lifestyle.

Filamentous fungi *A. flavus* produce many bioactive secondary metabolites, such as various mycotoxins or other bioactive compounds that have been exploited for pathogenicity. The genes responsible for the production of the secondary metabolites tend to be organized in biosynthetic gene clusters^49^. Using the whole genome of *A. flavus* JAM-JKB-BHA-GG20 as the query sequences of the antiSMASH 3.0 platform, many short and dense fragments present in some SM gene clusters, suggesting low prediction accuracy. Therefore, SM gene clusters were constructed.

From the present study, it can be summarized that the *Aspergillus flavus,* a most toxic and virulent fungal pathogen exist in Saurashtra region of Gujarat, which escort the threat of crashing down the export of groundnut seed market of this region in the future. This study identified fungal pathogen on basis of macroscopic, microscopic and molecular characters. The LC–MS QTOF demonstrated maximum presence of aflatoxin fractions B1, B2 than G1, G2 of total aflatoxin.

The whole genome data helped in understanding of aflatoxin gene pathway in comparison to other *Aspergillus* spp. and predicted presence of other secondary metabolites clusters viz*.* Nrps, T1pks etc*.* genes associated with biosynthesis of the OTA mycotoxin.

To get abdicated from such notorious fungal pathogen, an ecofriendly tool had been demonstrated by this study. The green AgNP’s formulated from the best antagonist bacterium were able to inhibit the most toxic and virulent fungus paving a new biological tool against a fungal pathogen and having capacity to hold down future economical crises in groundnut dependent families.

## Material and methods

### Extraction of aflatoxin using groundnut variety GG-20

The seeds of groundnut susceptible variety GG-20 were collected from Main Oil Seed Research Station, Junagadh Agricultural University, Gujarat. 10 g seed of GG-20 variety of groundnut were rehydrated with 10 ml of water, sterilized at 12 °C for 15 min, and inoculated with 1 ml of a spore suspension (approximately 6 × 10^–5^ spores/ml) from 21 isolates of a fungus^50^. The flasks were incubated at 28 °C for 5th, 10th and 15th day in an incubator with 80% relative humidity. After incubation for 5th, 10th and 15th day the seeds were crushed in 70% methanol and shaken at 150 rpm for 30 min at room temperature on a shaker in amber color 100 ml flasks and Filtered by Whatman filter paper. Samples filtrate were concentrated under N_2_ evaporator till it became to 2 ml and store in amber color 2 ml bottles until further used for aflatoxin detection by LCMS Q TOF. All methods were carried out in accordance with relevant guidelines by utilizing strict protocols as determined by the institute.

### Aflatoxin assay by LCMS Q TOF Analysis

Aflatoxin analyses were performed using an Agilent 6540UHD Accurate-Mass Q-TOF LC/MS^51^. The LC system consisted of a binary pump (G1312B), vacuum degasser (G1379B), a low carryover automatic liquid sampler (G1367D), thermostatted column compartment (G1316B) and Mass Hunter data system. Purified aflatoxin standards (B1, B2, G1 and G2) were obtained from Sigma-Aldrich and run 0.1–25 ppb of each simultaneously for quantifying aflatoxin fractions from unknown samples. Detailed procedure for aflatoxin assay using LCMS ids briefly highlighted in Additional File Text S2.

### Whole genome sequencing of highly virulent and toxigenic aflatoxin producing *A. flavus*

#### Isolation of genomic DNA

The fresh genomic DNA was isolated from seven day old culture grown on PDA medium at 28 °C^52^. Isolation of genomic DNA for whole genome sequencing was performed as described in Additional File Text S1.

#### Quantification and purity of genomic DNA

The genome DNA was quantified by Nanodrop machine and the concentration at A260/A280 was measured as described in supplementary material I. Pure DNA has an A260/A280 ratio of 1.8 in TE. DNA integrity was measure on 0.8% agarose gel electrophoresis. The concentration of DNA was adjusted to 100 ng µl^−1^ for further work.

#### Genome sequencing and assembly

Genome sequencing and assembly was performed into 3 steps i.e. 1: Library preparation; 2: Template preparation; 3: Sequencing. The detail of procedure employed is described in the Additional File Text S3.

#### Genome assembly and functional annotations

After quality control of the raw NGS data, the most toxic and highly virulent *Aspergillus flavus* JAM-JKB-BHA-GG20) genome assembly of reads was done by using CLC Genome Workbench 9. The draft genome was annotated by using the widely used AUGUSTUS version 2.7. To evaluate the potential of a gene to produce secondary metabolites, the assembled genome of the *A. flavus* was supplied to antiSMASH (v3.0.5, with default parameters, except for checking the “DNA of Eukaryotic Origin” box). RepeatMasker is program that screens DNA sequences for interspersed repeats and low complexity DNA sequence. Sequence comparisons in RepeatMasker were performed by ABBlast/WUBlast search engine. The Modeles of putative inter spread repeats were calculated by RepeatModeler which determined 416 repeat regions. The open reading frame (ORF) were identified by using programmed getorf details, which is part of EMBOSS package and tRNA’s were sorted out by using tRNA scans software.

### Comparative evaluation of aflatoxin producing predicted genes with other *Aspergillus* spp. genome

The genes predicted by software AUGUSTUS version 2.7 were converted into fasta format and the Basic Local Alignment Search (BLAST) analysis was carried with NCBI. The resulting hits were recorded to demonstrate percent identity and base pair coverage of test fungal genome with other *Aspergillus* spp.

### Dotplot analysis using aflatoxin specific gene sequences

Dotplot is a way to visualize the similarity between two protein or nucleic acid sequences^53^. Dotplots compare two sequences by organizing one sequence on the x-axis, and another on the y-axis, of a plot. When the residues of both sequences match at the same location on the plot, a dot is drawn at the corresponding position. To visualize the aflatoxin genes similarity within *Aspergillus* spp. a dotplot analysis was conducted. The aflatoxin genes derived from NCBI gene bank were utilized for creating dotplot.

### Ethical approval and consent to participate

The groundnut seeds varieties used in the proposed work were developed and released by Junagadh Agriculture University, India. Therefore, no specific permission was required to obtain seeds for research. Also, the work doesn’t involve any endangered or protected plant species so no further authorization is needed.

## Conclusion

In this study, we generated genome assembly of a highly toxic *Aspergillus flavus* isolate JAM-JKB-B HA-GG20. The whole genome data helped in understanding of aflatoxin gene pathway in comparison to other *Aspergillus* spp. and predicted presence of other secondary metabolites clusters viz Nrps, T1pks etc. genes associated with biosynthesis of the OTA mycotoxin. An aflatoxin producer isolate JAM-JKB-B HA-GG20 found morphologically similar to closely related non aflatoxigenic species, hence, will be useful for developing genetic screens to identify this toxic mold in crops and stored food products.

### Supplementary Information

Supplementary Information.

## Data Availability

All data discovered or designed in this study is available in this article and in Additional files. Raw data can be obtained from the corresponding author on reasonable request. Also the datasets generated during the current study are available in nucleotide database of NCBI with following accession number KU9859, KU984460, KU984461, KU984462, KU984463, KU984464, KU984465, KU984466, KU984467, KU984468, KU984469, KU984470, KU984471, KU984472, KU984473, KU984474, KU984475, KU984476, KU984477, KU984478, KU984479.
