# Supplementary material for: Whole genome sequencing and annotation of Aspergillus flavus JAM-JKB-B HA-GG20
Source: Sci Rep. 2024 Jan 2;14:18. doi: 10.1038/s41598-023-50986-5 (PMC10762212; doi:10.1038/s41598-023-50986-5)
Supplement: Supplementary file 1 — Supplementary Information. [file 41598_2023_50986_MOESM1_ESM.pdf]

Mass spectrum of toxigenic isolate producing B1 aflatoxin

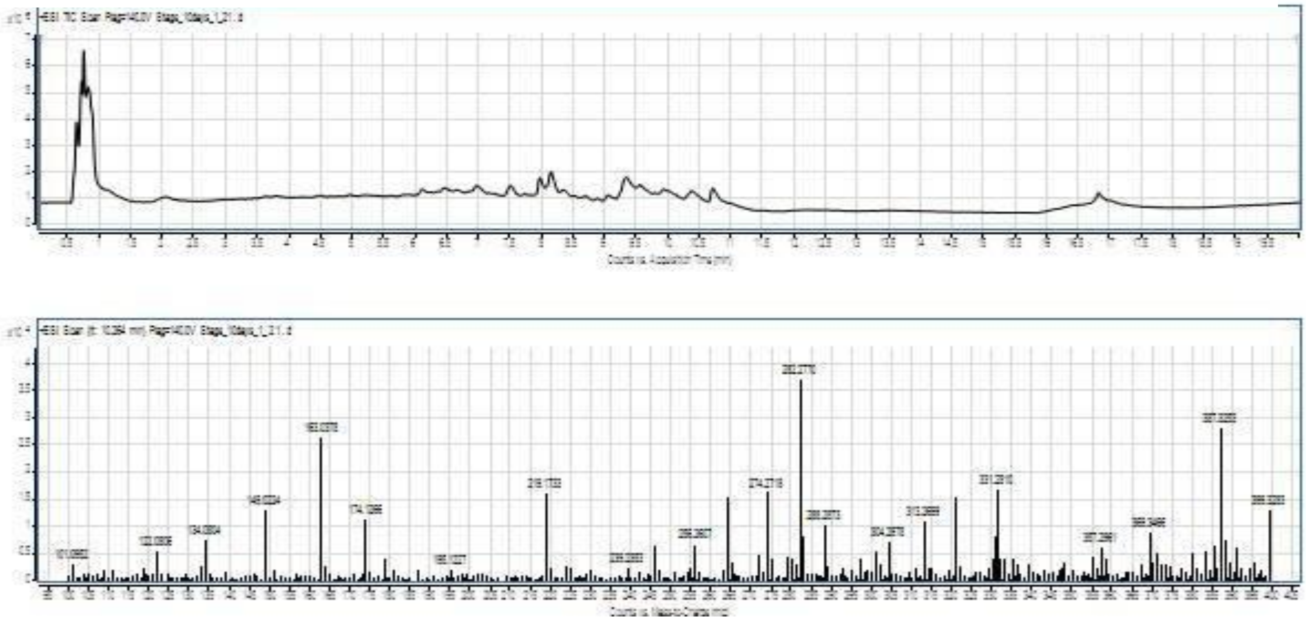

Mass spectrum of non toxigenic isolate producing B1 aflatoxin

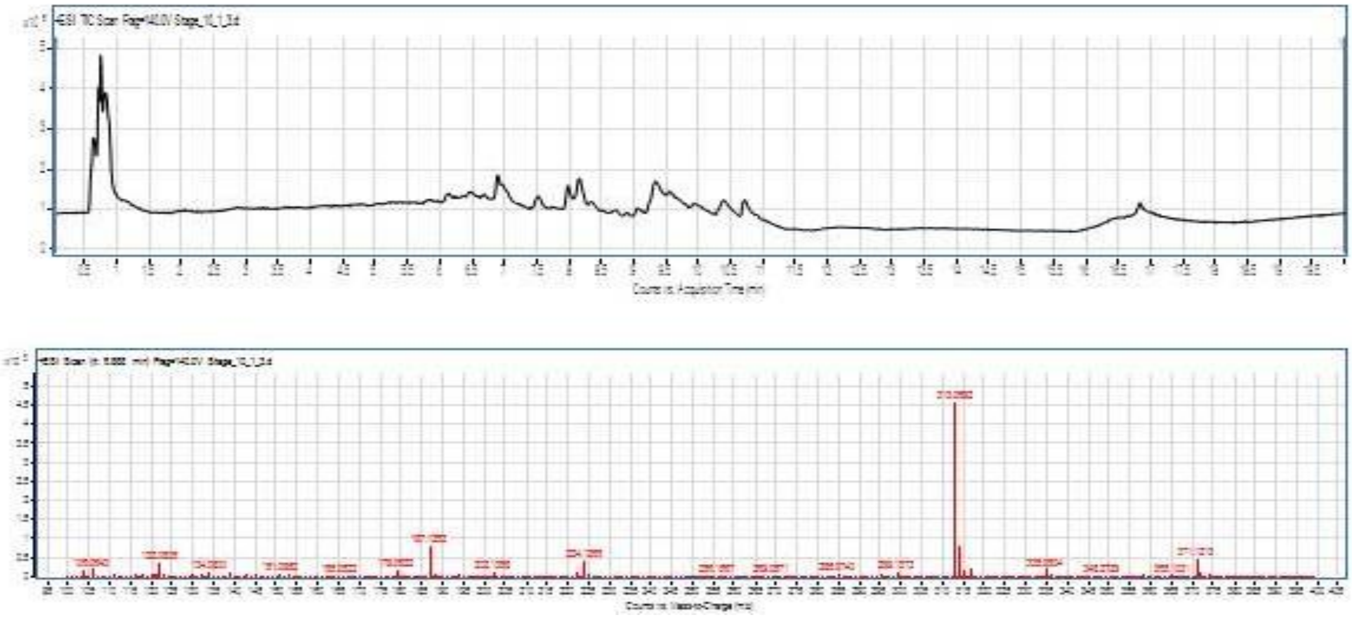

Figure S1A: Comparative chromatogram of toxigenic and non-toxigenic isolates producing B1 aflatoxin

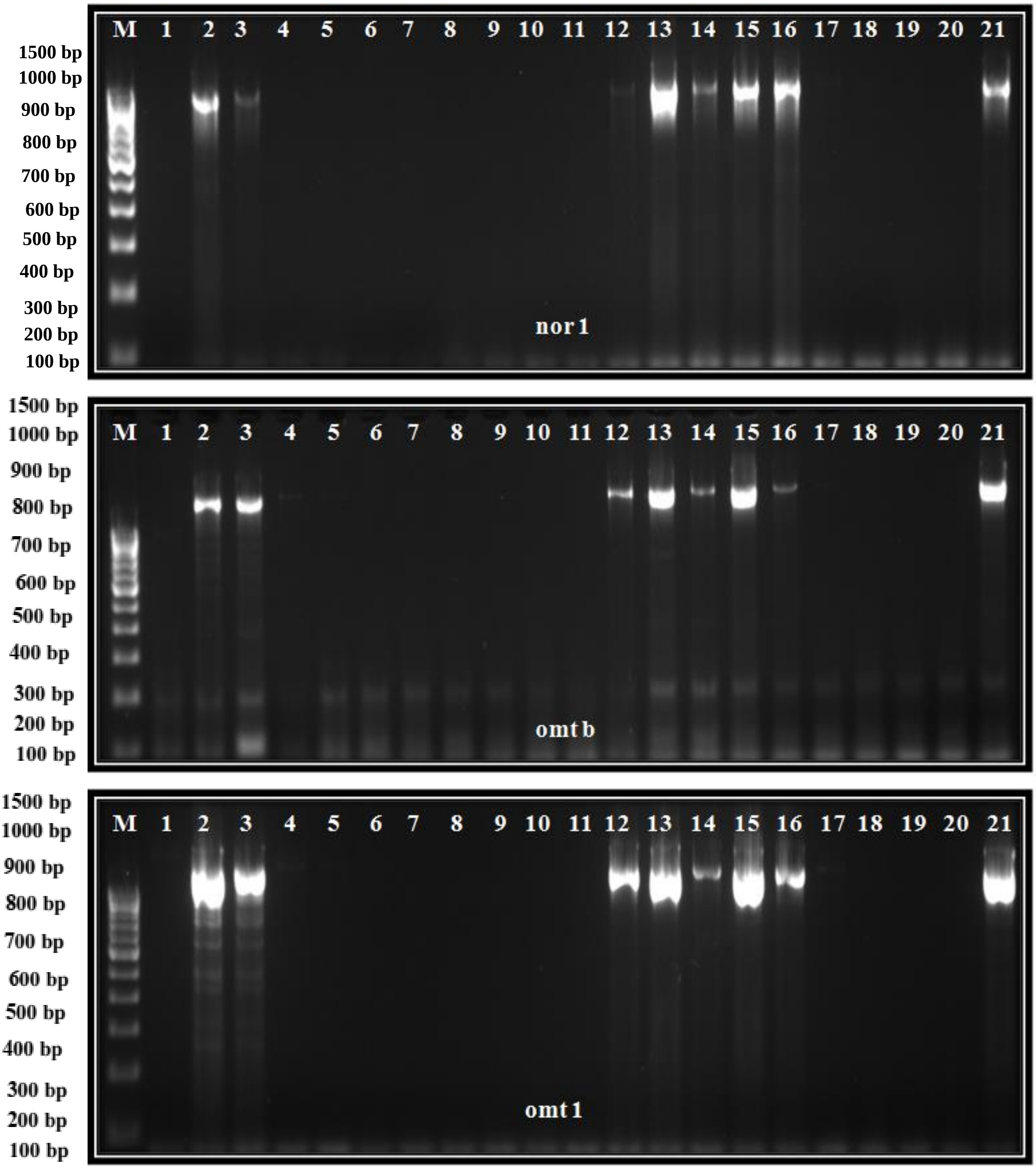

Figure S1B: Gene amplification profile confirmed aflatoxin producing and non-producing *Aspergillus* isolates derived from different parts of Saurashtra region with primer nor 1, omt b and omt 1. M=1 kb DNA ladder; 1: JND-VAD-VAD-GG45; 2: RJD-UPA-KUN-G-2; 3: JAM-JKB-BHA-GG20; 4: JND-MEN-MEN-GG41 A; 5: DWK-DWK-GG20 A; 6: JND-MEN-MEN-GG41 B; 7: UNA-YARD-3; 8: JND- YARD-2; 9: JND- YARD-1-GG20; 10: DWK-DWK-GG20 B; 11: JND-MEN-GUN-GG41; 12: RJD-UPA-KUN-TJ37A; 13: RJD-DHO-PAR-GG37; 14: RJD-DHO-KAN-GG45; 15: JND-MAN-LIM-GG20 A; 16: JND-MAN-LIM-GG20 B; 17: DAT-DAT-PAT-GG37; 18: JND-MEN-MEN-TJ45; 19: JND- JND-Cak-1; 20: JND- DGR -2036; 21: JND-DGR-1084. Gel images were photographed by Uvipro Gel documentation system using UVI gel start MW software version 11.1. Finally, the photographs were processed and text labeled in Adobe Photosop version 7.0.

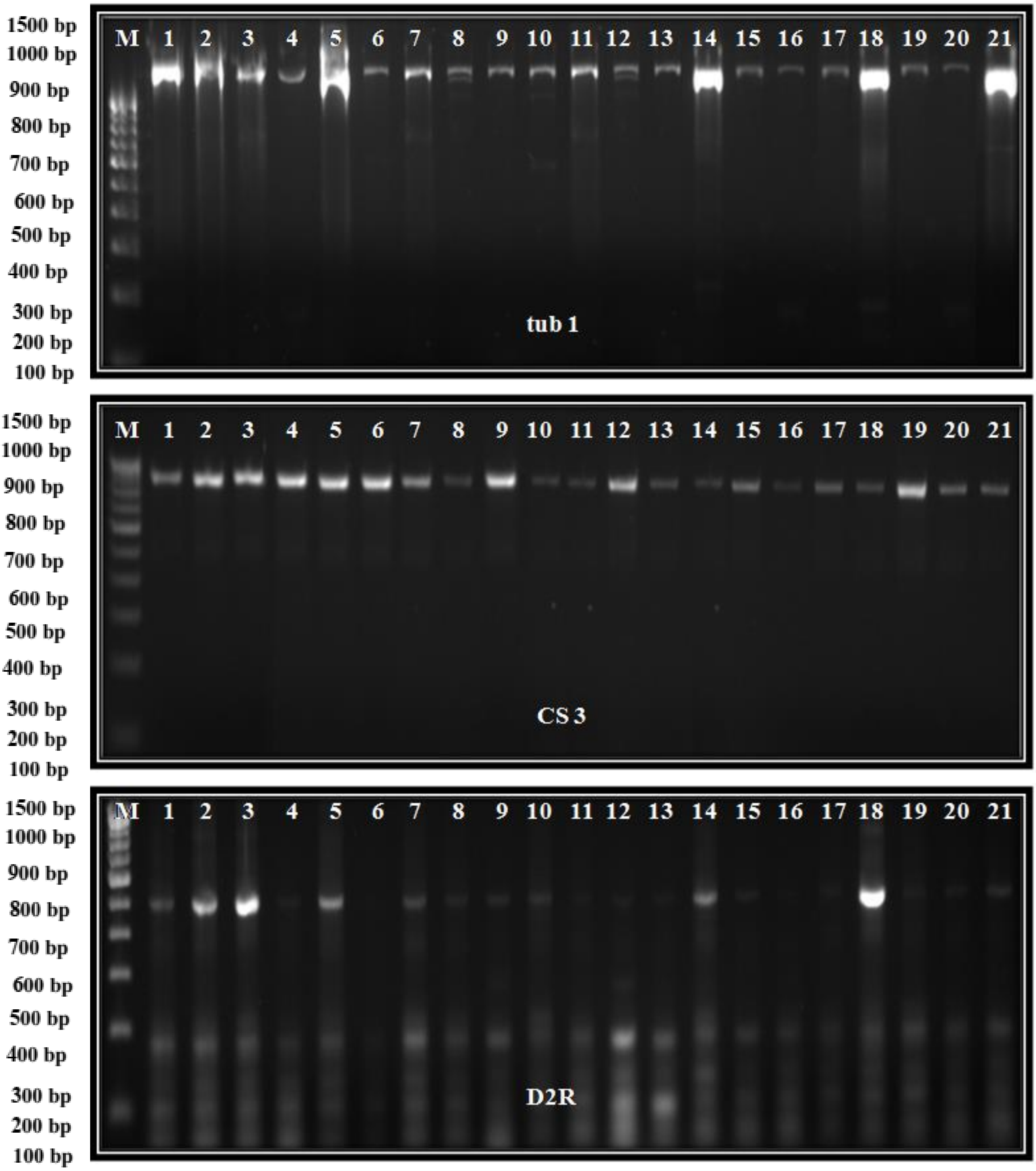

Figure S1C: PCR profile of β-tubulin gene and 28S rRNA region of fungus isolates isolated from different parts of Saurashtra region with primer tub 1, CS 3 and D2R. M=1 kb DNA ladder; 1: JND-VAD-VAD-GG45; 2: RJD-UPA-KUN-G-2; 3: JAM-JKB-BHA-GG20; 4: JND-MEN-MEN-GG41 A; 5: DWK-DWK-GG20 A; 6: JND-MEN-MEN-GG41 B; 7: UNA-YARD-3; 8: JND- YARD-2; 9: JND- YARD-1-GG20; 10: DWK-DWK-GG20 B; 11: JND-MEN-GUN-GG41; 12: RJD-UPA-KUN-TJ37A; 13: RJD-DHO-PAR-GG37; 14: RJD-DHO-KAN-GG45; 15: JND-MAN-LIM-GG20 A; 16: JND-MAN-LIM-GG20 B; 17: DAT-DAT-PAT-GG37; 18: JND-MEN-MEN-TJ45; 19: JND- JND-Cak-1; 20: JND- DGR -2036; 21: JND-DGR-1084. Gel images were photographed by Uvipro Gel documentation system using UVI gel start MW software version 11.1. Finally, the photographs were processed and text labeled in Adobe Photosop version 7.0.

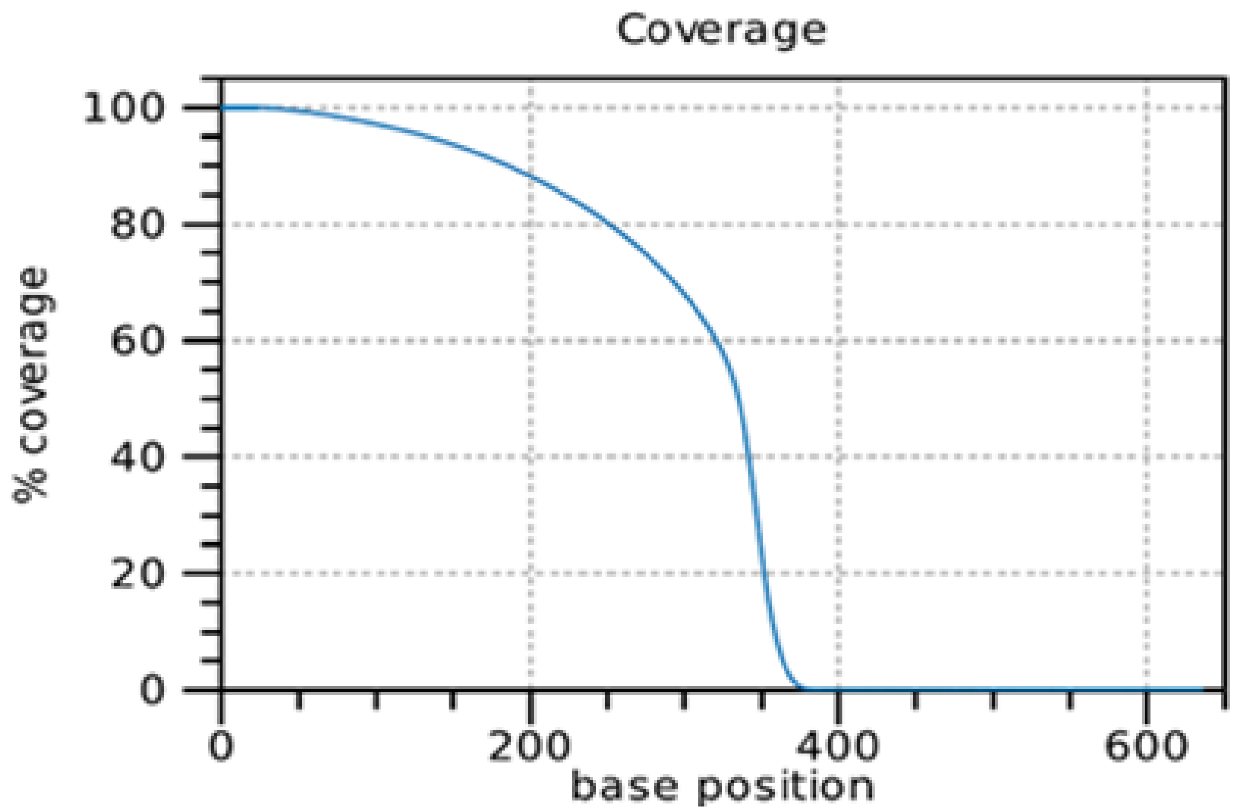

Figure S1D: The number of sequences that support (cover) the individual base positions. x: base position; y: number of sequences covering individual base positions normalized to the total number of sequences.

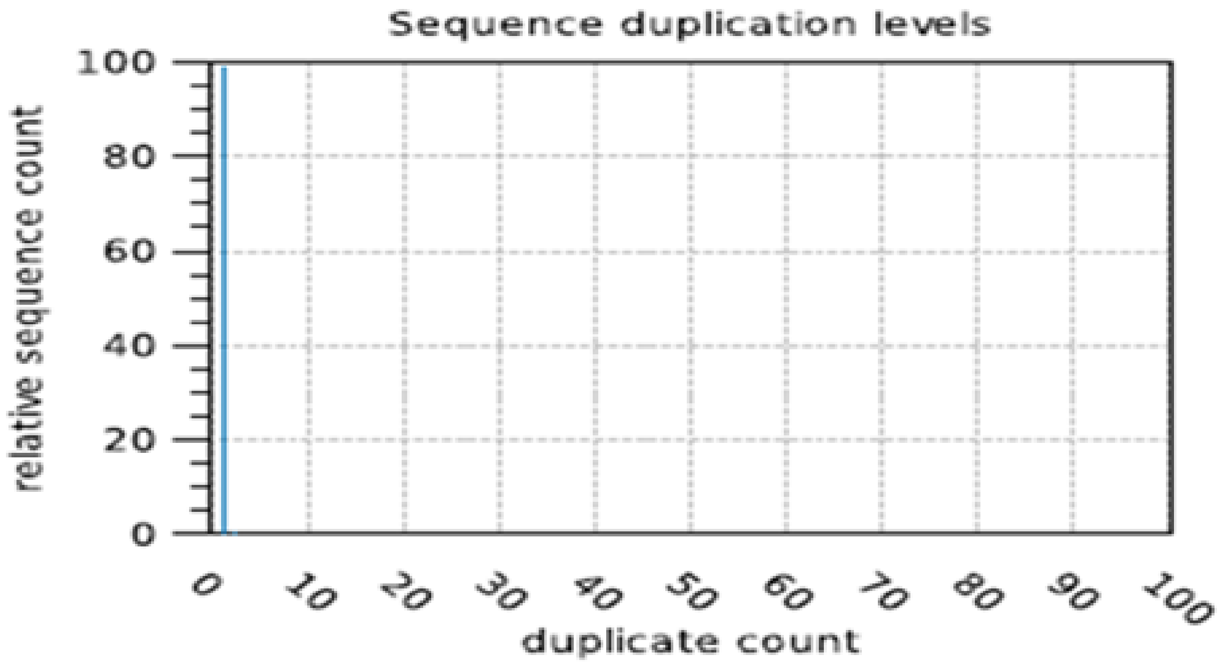

Figure S1E: Duplication level distribution. x: duplicate count; y: number of sequences that have been found that many times normalized to the number of unique sequence

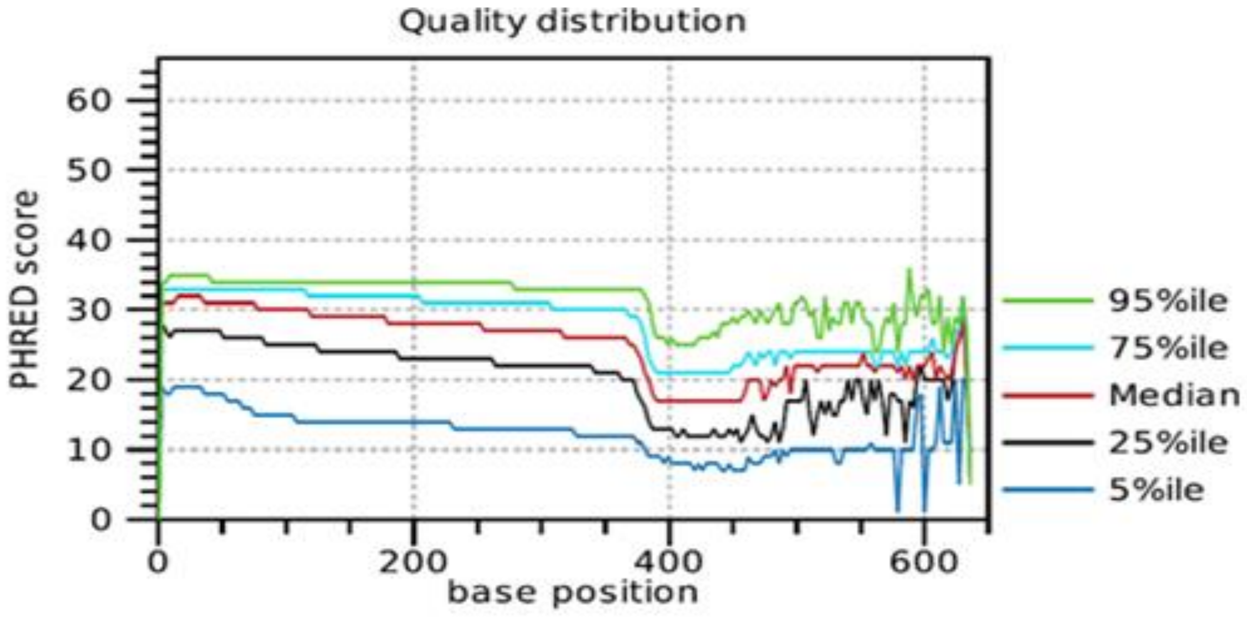

Figure S1F: Quality distribution of raw reads based on base quality distribution along the base positions. x: base position; y: median & percentiles of quality scores observed at that base position

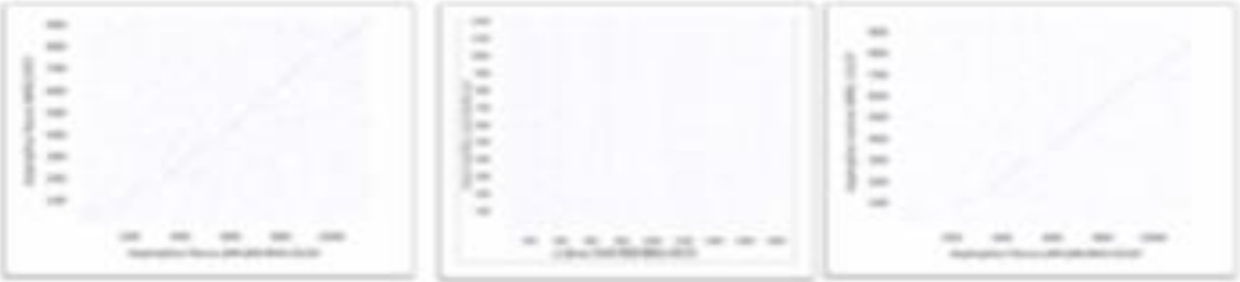

A. *ver-1* involved in the conversion of versicolorin A to sterigmatocystin

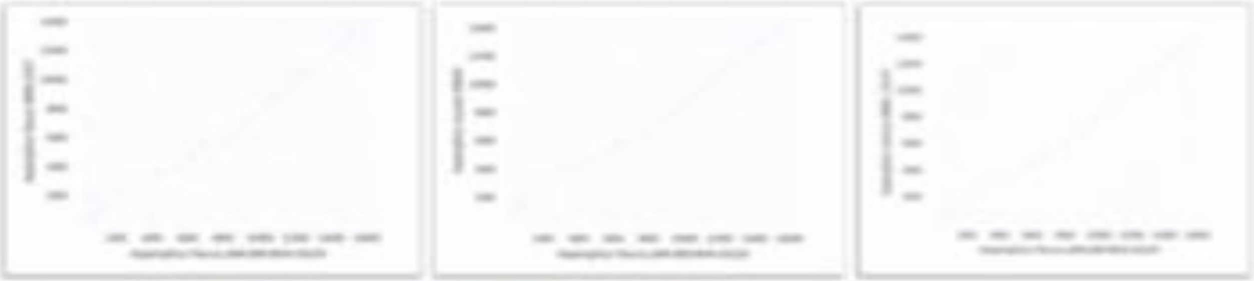

B. *pksA* gene, codes for a polyketide synthase

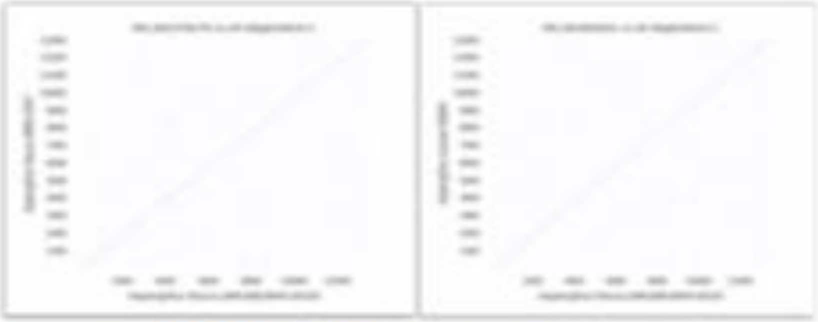

C. uvm8 gene involved in polyketide backbone synthesis

Plate 4: Dot plot comparisons of pairwise alignments of aflatoxin pathway genes viz. *nor-1*, *pksA*, *ver-1* and *Uvm8* of *A. flavus* JAM-JKB-BHA-GG20 with *A. flavus* NRRL 3357, *A. parasiticus* , *A. nomius* NRRL 13137, *A. oryzae* RIB 40 and *T. stipitatus* ATCC 10500

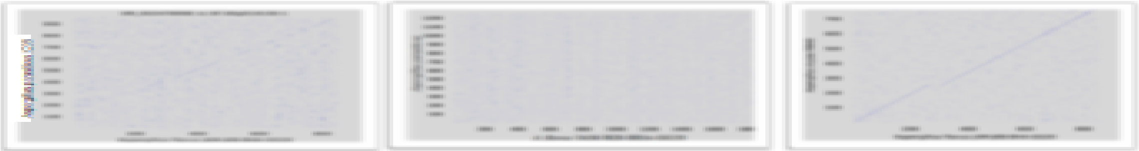

D. *nor-1* gene, which codes for a reductase that converts norsolorinic acid to averantin

Plate 5: Dot plot comparisons of pairwise alignments of aflatoxin pathway genes viz. *nor-1*, *pksA*, *ver-1* and *Uvm8* of *A. flavus* JAM-JKB-BHA-GG20 with *A. flavus* NRRL 3357 and *A. oryzae* RIB 40

Figure S1G: Dot plot analysis among aflatoxin genes in *Aspergillus*

Table S1: Colony morphology of *Aspergillus* strains on PDA and YES matrix

Isolate No.	Isolate code	Type of Media	Colony color	Colony reverse color	Colony edge	Mycelial color	Conidiation	Shape of conidia	Conidial wall	Mycelium Growth (mm)		Growth rate
										3 day	7 day	
1	JND-VAD-VAD-GG45	PDA	Yellowish Green	Orange	Smooth	White	Circular	Round	Smooth	35	79	Medium
		YES	Creamiest Brown	Cream	Smooth	White	Spot ring Like flat	Round	Smooth	90	90	Fast
2	RJD-UPA-KUN-G2	PDA	Green	Yellow	Smooth	White	Spot ring Like flat	Round	Smooth	90	90	Fast
		YES	Yellowish Green	Yellow	Smooth	White	Spot ring Like flat	Round	Smooth	75	90	Fast
3	JAM-JKB-BHA-GG20	PDA	Green	Cream	Smooth	White	Spot ring Like flat	Round	Smooth	50	90	Medium
		YES	Yellowish Green	Light Orange	Smooth	White	Spot ring Like flat	Round	Smooth	65	90	Fast
4	JND-MEN-MEN-GG41	PDA	Greenish brown	Cream	Smooth	White	Spot ring Like flat	Round	Smooth	35	90	Medium
		YES	Greenish Brown	Cream	Smooth	White	Spot ring Like flat	Round	Smooth	90	90	Fast
5	DWK-DWK-GG20	PDA	Green	White	Smooth	White	Spot ring Like flat	Round	Smooth	45	80	Medium
		YES	Yellowish Green	Light Orange	Smooth	White	Spot ring Like flat	Round	Smooth	40	80	Medium
6	JND-MEN-MEN-GG41	PDA	Greenish Brown	White	Smooth	White	Spot ring Like flat	Round	Smooth	65	90	Medium
		YES	Brown	Cream	Smooth	White	Spot ring Like flat	Round	Smooth	45	90	Medium

7	JND-MISC-3	PDA	Green	White	Smooth	White	Spot ring Like flat	Round	Smooth	40	90	Medium
		YES	Yellowish Green	Cream	Smooth	White	Circular	Round	Smooth	60	90	Fast
8	JND-MISC-2	PDA	Greenish Brown	White	Smooth	White	Spot ring Like flat	Round	Smooth	60	90	Medium
		YES	Yellowish Green	Yelloe	Smooth	White	Spot ring Like flat	Round	Smooth	90	90	Fast
9	JND- YARD-1-G	PDA	Green	White	Smooth	White	Spot ring Like flat	Round	Smooth	90	90	Fast
		YES	Brownish Green	Light Orange	Smooth	White	Spot ring Like flat	Round	Smooth	55	90	Fast
10	DWK-DWK-GG20	PDA	Brown	White	Smooth	White	Spot ring Like flat	Round	Smooth	55	90	Medium
		YES	Brown	Yellow	Smooth	White	Spot ring Like flat	Round	Smooth	50	90	Medium
11	JND-MEN-GUN-GG41	PDA	Brown	White	Smooth	White	Spot ring Like flat	Round	Smooth	45	75	Medium
		YES	Brown	Cream	Smooth	White	Circular	Round	Smooth	60	90	Fast
12	RJD-UPA-KUN-TJ37A	PDA	Green	Yellow	Smooth	White	Spot ring Like flat	Round	Smooth	90	90	Fast
		YES	Yellowish Green	Yellow	Smooth	White	Spot ring Like flat	Round	Smooth	70	90	Fast
13	RJD-DHO-PAR-GG37	PDA	Dark Green	Yellow	Smooth	White	Circular	Round	Smooth	55	90	Slow
		YES	Yellowish Green	Yellow	Smooth	White	Circular	Round	Smooth	55	90	Slow
14	RJD-DHO-KAN-GG45	PDA	Green	Yellow	Smooth	White	Spot ring Like flat	Round	Smooth	50	90	Medium
		YES	Green	Whitish Orange	Smooth	White	Spot ring Like flat	Round	Smooth	55	90	Fast

15	JND-MAN-LIM-GG20	PDA	Dark Green	Yellow	Smooth	White	Circular	Round	Smooth	50	90	Slow
		YES	Green	Red	Smooth	White	Spot ring Like flat	Round	Smooth	90	90	Fast
16	JND-MAN-LIM-GG20	PDA	Dark Green	Yellow	Smooth	White	Circular	Round	Smooth	65	90	Slow
		YES	White	Orange	Smooth	White	Circular	Round	Smooth	40	55	Slow
17	DAT-DAT-PAT-GG37	PDA	Brown	Cream	Smooth	White	Spot ring Like flat	Round	Smooth	55	75	Medium
		YES	White	Orange	Smooth	White	Circular	Round	Smooth	45	55	Slow
18	JND-MEN-MEN-TJ45	PDA	Green	White	Smooth	White	Spot ring Like flat	Round	Smooth	90	90	Fast
		YES	Greenish Yellow	Orange	Smooth	White	Spot ring Like flat	Round	Smooth	50	90	Medium
19	JND- JND-Cak-1	PDA	Green	White	Smooth	White	Spot ring Like flat	Round	Smooth	30	85	Medium
		YES	Greenish Yellow	Orange	Smooth	White	Spot ring Like flat	Round	Smooth	90	90	Fast
20	JND- JAU – HSS-1	PDA	Brown	Cream	Smooth	White	Spot ring Like flat	Round	Smooth	30	70	Medium
		YES	Brown	Cream	Smooth	White	Spot ring Like flat	Round	Smooth	90	90	Fast
21	JND- JAU – ISS-1	PDA	Green	Light Green	Smooth	White	Circular	Round	Smooth	50	90	Medium
		YES	Yellow	Light Brown	Smooth	White	Circular	Round	Smooth	40	90	Medium

Supplementary Text File S1

LCMS Q TOF Analysis Instrumentation

Rapid resolution and configuration

- Agilent 1200 Series Binary Pump SL (G1312B)
- High Performance WP Sampler SL Plus (G1367D) Sampler Thermostat (G1330B)
- Thermostatted Column Compartment SL, including 10P/Two-Position switching valve (G1316B with option #057)

Method Conditions

Column	:	Agilent ZORBAX Eclipse Plus C18, 2.1 x 50 mm, 1.8 µm		
Column temperature	:	40 °C		
Sample Injection volume	:	10 Ml		
Auto sampler temperature	:	4 °C		
Needle wash	:	Flushport (100% methanol), 5 seconds		
Mobile phase	:	A = 10 mM NH4 acetate in water<br>B = 100% methanol		
Gradient flow rate	:	0.6 mL/min (no split)		
Gradient	:	Time (min)	A %	B %
		0:00<br>8:00<br>14:00<br>15:00<br>20:00	90<br>00<br>00<br>90<br>90	10<br>100<br>100<br>10<br>10
Flow /min		0.8 ml		
Total run time	:	20 min		
Pressure	:	171 to 600 bar		

The Triple Quadrupole MS parameters are listed in table 3.4. All fragment or voltage settings and respective collision energies (CE) and the most abundant MS/MS product ions per analyte were determined automatically using the Agilent Mass Hunter Optimizer Software

The Triple Quadrupole MS parameters

**Name**	**Retention time (min)**	**Precursor ion (m/z)**
Aflatoxin B1	4.68	313.0709
Aflatoxin B2	4.57	315.0867
Aflatoxin G1	4.40	329.0656
Aflatoxin G2	4.26	331.0812

A typical chromatogram is shown in Fig. 3.1, which illustrates ppb concentration level of each aflatoxin together with the corresponding time in minutes. Standard curves for aflatoxins B1, B2, G1 and G2 all showed a good linearity through the concentration range 0.1 to 100 ppb each with a linear correlation (R2) of greater than 0.999. Fig. 3.2 illustrates an overlay of each standard curve on the same scale.

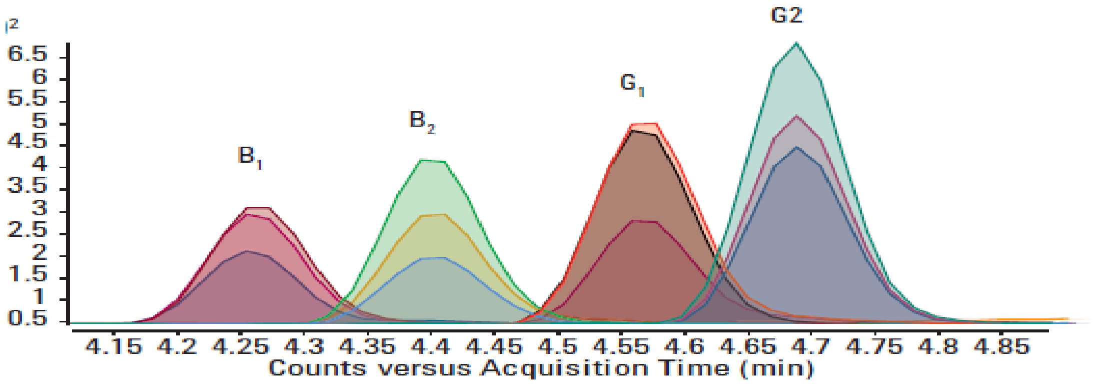

Chromatographic resolution for each aflatoxin B1, B2, G1 and G2

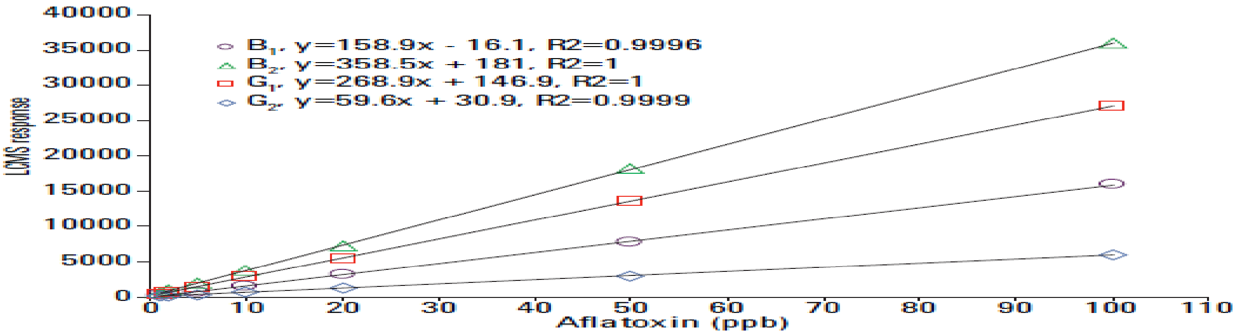

Overlaid standard curves for aflatoxins B1, B2, G1 and G2

Supplementary Text File S2

DNA Isolation Procedure Amer *et al.* (2011)

1. Ground 1 g (fresh weight) of mycelium into a fine powder in a mortar pre-cooled at -80 ºC.
2. Suspend powder in 1.5 ml of lysis buffer.
3. Incubate it at 68 ºC for 15 minutes, with occasional gentle mixing.
4. Centrifuge sample at 13, 000 rpm for 15 minutes (4 ºC).
5. Transfer the supernatant to a new tube
6. Add 750 µl of cold 4 M sodium acetate, at pH 5.2 with gently mixing by inversion.
7. Incubate at -20 ºC for 20 minutes.
8. Centrifuged at 13, 000 rpm for 15 minutes (4ºC).
9. Transfer the supernatant to a new tube
10. Add equal volume of cold isopropanol (-20 ºC) and incubate at -20 ºC for 10 minutes.
11. Centrifuge sample at 13, 000 rpm for 15 minutes (4 ºC).
12. Decant the supernatant and wash the pellet with 70% ethanol by centrifugation at 10,000 rpm for 10 min.
13. Decant the supernatant and air dry the pellet at room temperature.
14. Suspend the pellet in 100 µl of TE.

Quantification and purity test of DNA

To estimate the quantity and quality (in terms of protein and RNA contamination) of isolated genomic DNA, spectrophotometry was performed by using software N.D. spectrophotometer (V.3.3.0). Three µl DNA was loaded into the well of Nanodrop machine and the concentration at A260/A280 was measured. Pure DNA has an A260/A280 ratio of 1.8 in TE. Strong absorbance at 280 nm, resulting in a low A260/A280 ratio, indicates the presence of contaminants such as proteins. The concentration of DNA was adjusted to 50 ng.μl^{-1} for further work.

Supplementary Text File S3

1) Library preparation 2) Template preparation and 3) Sequencing.

Library preparation (Fragment gDNA with Ion Shear™ Plus Reagents)

Library was prepared by enzymatic degradation of DNA into blunt ended fragments which was further used for adapter ligation. The fragmentation and purification of DNA was done by using Ion Xpress Plus Fragment library kit provided by invitrogen.

Materials and equipment required

**Materials provided in the Ion Xpress™ Plus Fragment Library Kit**	**Other materials and equipment required**
Ion Shear™ Plus 10X Reaction Buffer	Nuclease-free Water
Ion Shear™ Plus Enzyme Mix II	1.5ml Eppendorf® LoBind tubes
Ion Shear™ Plus Stop Buffer	0.2ml PCR tubes
Low TE	37°C heat block/water bath
	P10–P20 and P100–P200 pipettors
	Agencourt® AMPure® XP Kit

Protocol for DNA fragmentation

1. The concentration of gDNA was adjusted to 100ng/µl in nuclease free water.
2. The 5 µl of Ion shear plus 10X reaction buffer was added in lobind 1.5ml tube with 10 µl of gDNA (100ng/µl) along with 25 nuclease free water.
3. Add 10µl Ion shear plus enzyme mix-II in the new tube to make the reaction mixture 50 µl.
4. The reaction was mixed by rapidly pipetting up and down 8–10 times.
5. The tube was incubated in a water bath at 37°C for 15 min. to fragment 200-300bp gDNA.
6. Stop buffer was immediately added 5µl of Ion Shear™ after incubation and mixed thoroughly by vortexing for at least 5 seconds. Stored the reaction tube on ice.

Purification of fragmented DNA

1. Add 99µl of Agencourt® AMPure® XP Reagent (1.8X sample volume) to the sheared DNA sample by thorough mix the bead suspension with the DNA by pipette up and down 5 times, then pulse-spined and incubated the mixture at room temperature for 5 minutes.
2. The tube was placed in a magnetic rack after Pulse-spined in such as the DynaMag™-2 magnet for 3 minutes or until the solution was cleared of brown

tint when viewed at an angle. Carefully removed and discarded the supernatant without disturbing the bead pellet.

3. Without removing the tube from the magnet, added 500μl of freshly prepared 70% ethanol. Incubated for 30 sec., turned the tube around twice in the magnet to move the beads around. After the solution became cleared, removed and discarded the supernatant without disturbed the pellet.
4. Step 3 was repeated for a second wash.
5. Remove residual ethanol by pulse-spining the tube, placed it back in the magnetic rack and carefully removed any remained supernatant with a 20μl pipettor without disturbing the pellet.
6. The tube was air-dryed by keeping the beads on the magnet at room temperature for ≤5 minutes.
7. The tube was removed from the magnetic rack and 25μl of Low TE was directly added to the pellet to disperse the beads. Pipetted the suspension up and down 5 times then vortexed the sample for 10 sec. to mix thoroughly.
8. The tube was placed in the magnetic rack for at least 1 min. after Pulse-spined until the solution cleared. Transferred the supernatant contained the eluted DNA to a new 0.2ml PCR tube without disturbed the pellet.

Ligation of adapters, nick repair and purify the ligated DNA

For ligation of adapter, nick repair and purify the ligated DNA following materials was used.

Materials and Equipment

**Materials provided in the Ion Plus Fragment Library Kit**	**Other materials and equipment required**
10X Ligase Buffer	0.2ml PCR tubes
Adapters	Thermal cycler
DNA Ligase	Nuclease-free Water
Nick Repair Polymerase	Agencourt® AMPure® XP Kit
dNTP Mix	Freshly prepared 70% ethanol

Reaction mixture for barcoded libraries

Sr. No.	Component	Volume by input DNA	
1.	DNA	~25µl	~25µl
2.	10X Ligase Buffer	10µl	10µl
3.	Ion P1 Adapter	2µl	10µl
4.	Ion Xpress™ Barcode	2µl	10µl
5.	dNTP Mix	2µl	2µl
6.	Nuclease-free Water	49µl	31µl
7.	DNA Ligase	3µl	5µl
8.	Nick Repair Polymerase	8µl	8µl
**Total**		**100µl**	**100µl**

PCR condition for barcoded libraries

Sr. No.	Stage	Temp.	Time
1.	Hold	25°C	15 min
2.	Hold	72°C	5 min
3.	Hold	4°C	∞

Protocol

1. In a 0.2ml PCR tube, the reagents as indicated (Table 3.15) was combined for non-barcoded and mixed well by pipetting up and down.
2. Placed the tube in a thermal cycler and run the following program. Transfer the entire reaction mixture to a 1.5ml Low bind tube for the next cleanup step (Table 3.16).
3. For purification, the 140µl volume of Agencourt® AMPure® XP reagent was added to the sample, pipetted up and down 5 times to thoroughly mixed the bead suspension with the DNA, pulse-spined the tube and incubated the mixture for 5 minutes at room temperature.
4. The tube was placed in a magnetic rack the DynaMag™2 magnet for 3 min after Pulse-spin. or until the solution was cleared. Carefully removed and discarded the supernatant without disturbing the pellet.
5. Without removing the tube from the magnet, 500µl of freshly prepared 70% ethanol was added. Incubated for 30 seconds, turned the tube around twice in

the magnet to move the beads around. After the solution became clear, removed and discarded the supernatant without disturbing the pellet.

6. For a second wash step 3 was repeated.
7. Remove residual ethanol by pulse-spined the tube, placed it back in the magnetic rack, and carefully removed any remaining supernatant with a 20µl pipettor without disturbing the pellet.
8. The tube was kept on the magnetic rack, air-dry the beads at room temperature for ≤5 minutes.
9. The tube from the magnetic rack removed and 20µl of low TE directly added to the pellet to disperse the beads. Pipette the mixture up and down 5 times then vortexed the sample for 10 seconds, to mix thoroughly.
10. The tube were placed in the magnetic rack for at least one min. after Pulse-spin until the solution became clear. Transferred the supernatant which contained the eluted DNA to a new 1.5ml Low bind tube without disturbing the pellet.

Size-selection of DNA fragment

Size-selection of DNA fragment was done by E-Gel® SizeSelect™ Agarose Gel.

Materials and equipments required

E-Gel® iBase™ unit and E-Gel® Safe Imager™ transilluminator combo kit	300-base-read libraries: 100-bp DNA ladder
E-Gel® SizeSelect™ 2% agarose gel	Nuclease-free water
100- or 200-base-read libraries: 50-bp DNA Ladder	

Protocol

1. 250ng of DNA ladder was loaded (50-bp DNA ladder for 100- or 200-base-read libraries.
2. For 1µg input samples, 20µl of low TE was added to the purified ligated DNA to bring the total volume to 20µl.
3. Add 20 µl of ligated DNA to the loading well (top row).
4. Twenty-five µl of nuclease-free water was added to all empty wells in the top row and add 10µl to the center nell (lane M) bottom row.

5. Placed the amber filter over the E-Gel® iBase™ unit. Selected run size-select 2% program and run the gel for 12-14 min. and target 330bp.
6. Pressed go on the iBase™ unit to start electrophoresis. The red light turned to green.
7. Monitored the appropriately sized ladder band to the reference line with periodic monitoring of the run.
8. To stop the run pressed go again when the band reached the reference line.
9. The collection wells were refilled to 25µl with ~10µl of nuclease-free water. The water in the wells could form a concave surface.
10. The solution was collected from the collection wells using a pipette, without piercing the bottom of the well.

Amplification and purification of library

Materials and equipments

Platinum® PCR superMix high fidelity	Library amplification primer mix
Thermal cycler	Agencourt® AMPure® XP kit
0.2ml PCR tubes	Freshly prepared 70% ethanol
1.5ml Low bind tubes	Magnetic rack

a) Protocol for library amplification

1. Adjusted the volume of the unamplified library as described below.

**Size-selection method**	**E-Gel® SizeSelect™ Agarose Gel**
Library input amount	50–100ng
Volume, unamplified library	~30ml
Volume to amplification rxn (step 2)	25ml

2. The following reagents were combined in an appropriately sized tube and mixed by pipetting up and down.

Reaction mixture for library amplification

**Sr. No.**	**Component**	**Volume input by DNA**
		**50-100ng**
**1**	Platinum® PCR super mix high fidelity	200µl
**2**	Library amplification primer mix*	5µl
**3**	Unamplified library	25µl
Total		130µl

3. Reaction mixture was splited 130µl into two 0.2ml PCR tubes, each containing about 65µl.
4. The tubes were placed in to a thermal cycler and run the following PCR cycling program.

Reaction condition for library amplification

Sr. No.	Step	Temp.	Time
1.	Initial Denaturation	95°C	5 min
2.	Denaturation	95°C	15 sec
3.	Annealing	58°C	15 sec
4.	Extension	70°C	1 min
Repeat the steps 2 to 4 for 10 Cycles			
5.	Hold	4°C	∞

5. Previously split PCRs was combined in a new 1.5ml Low bind tube.

b) Procedure for purification of library

1. 195µl Agencourt® AMPure® XP reagent (1.5X sample volume) was added to each sample.
2. Pipette up and down 5 times to thoroughly mixed the bead suspension with the DNA, then pulse-spined and incubated the mixture for 5 min. at room temperature.
3. The tube was placed in a magnetic rack such as the DynaMag™-2 magnet for three minutes or until the solution was cleared. Carefully removed and discarded the supernatant without disturbing the pellet.
4. Without removing the tube from the magnet, 500µl of freshly prepared 70% ethanol was added. Incubated for 30 sec. and turned the tube around twice in the magnet to move the beads around. After the solution was cleared, removed and discarded the supernatant without disturbed the pellet.
5. For second wash step 4 was repeated.
6. Residual ethanol was removed by pulse-spined the tube, placed it back in the magnetic rack and carefully removed any remained supernatant with a 20µl pipettor without disturbed the pellet.
7. The tubes were kept on the magnet, air-dry the beads at room temperature for ≤5 min.

8. Removed the tube from the magnetic rack and 20µl of low TE was directly added to the pellet to disperse the beads. Pipette the mixture up and down five times then vortexed the sample for 10 seconds, to mix thoroughly.
9. Pulse-spined and placed the tube in the magnetic rack for at least 1 min. until the solution became clear. Transferred the supernatant without disturbing the pellet in a new 1.5ml low bind tube which contained the eluted DNA.
10. Residual beads was removed from the eluted DNA by placed the tube with the eluted DNA back on the magnet for at least one minute and transferred the supernatant to a new 1.5ml low bind tube without disturbed the pellet (store the library at –30°C to –10°C).

Template preparation

After the dilution of library by the template dilution factor, used 20µl of the diluted library was used in the amplification reaction, which was loaded on the Ion OneTouch™ Instrument.

Materials and other equipments required

**Provided in the Ion OneTouch™ 200 solutions kit v2**	**Provided in the Ion PGM™ control materials kit**
• Ion OneTouch™ Reaction Oil (small bottle)	• *E. coli* DH10B Control 200 Library
**Provided in the Ion OneTouch™ 200 Reagents Kit v2**	**Other materials and equipments**
• Ion OneTouch™ enzyme mix	• Diluted library
• Ion OneTouch™ 2X reagent mix	• Microcentrifuge
• Ion OneTouch™ 200 Ion Sphere™ Particles	• 1.5ml low binding Tubes
**Provided in the Ion OneTouch™ 200 Reactions Kit v2**	• Tube rack to fit 15ml conical tube
• Ion OneTouch™ Plus reaction filter assembly	• Vortexer

Preparation of amplification solution

1. The reagents were prepared as followed.

**Reagents**	**Preparation**
Ion OneTouch^{Tm}2X Reagent mix	This fully thawed solution vortexed for 30 sec. and centrifuged for 2 mins. Kept it at room temp.

Ion OneTouch^{Tm}Enzyme Mix	Centrifuged the enzyme for 2 sec. and placed it on ice.
Library	Vortexed the solution for 5 sec. and then centrifuged the solution for 2 sec.

2. In a 1.5ml low bind tube at 15°C to 30°C, added the following components in the designated order. Each component was added and then pipetted the amplification solution up and down to mix.

Order	Reagent	Volume
**1**	Nuclease-free water	280µl
**2**	Ion OneTouch^{Tm}2X reagent mix	500µl
**3**	Ion OneTouch^{Tm} Enzyme mix	100µl
**4**	Diluted library	20µl
Total		900µl

3. The Ion Sphere Particles (ISP's) was Vortexed at maximum speed for one minute to resuspend the particles, pipetted the ISPs up and down to mix, following by addition of the ISPs to the amplification solution.

Order	Reagent	Volume
**1**	Amplification solution without ISP (from step 2 of this procedure)	900µl
**2**	Ion OneTouch^{Tm}200 Ion Sphere Particles	100µl
Total		1000µl

Adding the amplification solution through the sample port

1. P1000 pipette was set to 1000µl, and attached a new 1000µl tip to the pipette.
2. The amplification solution was vortexed at maximum speed for 5 seconds, then centrifuged the solution for 2 seconds.
3. The amplification solution was pipetted up and down to mix and then filled the tip with 1000µl of the amplification solution.
4. Inserted the tip firmly into the sample port so that the tip was perpendicular to the Ion OneTouch™ plus reaction filter assembly and fully inserted into the sample port to form a tight seal.
5. Slowly pipette the entire 1000µl of the amplification solution through the sample port and then kept the plunger of the pipette depressed.
6. The tip from the sample port was removed and then appropriately discarded the tip.

7. Add Ion OneTouch™ reaction oil through the sample port.

Installation of filled Ion OneTouch™ plus reaction filter assembly on the Ion OneTouch™ DL configuration

1. Ion OneTouch™ plus reaction filter assembly was kept in the tube rack; orient the assembly so that the sample port is on left.
2. Lifted straight out the Ion OneTouch™ plus reaction filter assembly from the tube rack. With the short tubing in the reaction tube on the left, rotate the assembly to right until the reaction tube was inverted and the three ports of the reaction plus filter face down.

Running of Ion OneTouch™ instrument DL configuration

1. Ensured that the centrifuge lid of the instrument was closed.
2. On the home screen, touched run.
3. To choose the run type, touched assisted**.**

ISPs Materials required for recover the template-positive

**Provided in the Ion OneTouch™ 200Solutions Kit v2**	**Other materials and equipments**
Ion OneTouch™ Wash Solution	1.5ml low bind tubes
	Pipettes
	Vortexer
	Microcentrifuge

Protocol

1. At the end of the run, ensured to centrifuged the samples.
2. Immediately after the centrifuge stops, removed and discarded the recovery router.
3. Carefully removed both recovery tubes from the instrument and put the two recovery tubes in a tube rack. If seen some cloudiness in the tube, which was normal and washed the template-positive ISPs.

Genome sequencing with Ion torrent (Life technologies) S5 platform.

Loading of chip

1. Tilt the chip 45 degrees so that the loading port was at the lower port.

2. The pipette tip was inserted firmly into the loading port and removed as much liquid as possible from the loading port. Discarded the liquid.
3. The chip was placed upside-down in the centrifuge adapter bucket and transfer the bucket to the mini fuge with the chip tab pointing in (toward the center of the mini fuge).
4. The chip was completely empty by five seconds centrifugation. Removed the chip from the bucket and wiped off any liquid from the bucket.
5. The chip was transferred to the mini fuge with the chip tab pointing in (toward the center of the mini fuge).
6. Remove the chip from the centrifuge bucket after centrifuging for 30 seconds.
7. The sample was mixed in the chip by following the below step:
    a. Set the pipette volume to 30µl.
    b. Tilt the chip 45 degrees so that the loading port was at the lower port, and inserted the pipette tip into the loading port.
    c. Without removing the tip, slowly pipette the sample in and out of the chip three times. Pipette slowly to avoid creating bubbles.
8. The chip was centrifuged for 30 seconds with the chip tab pointing out (away from the center of the mini fuge).
9. The chip mixing was repeated in step 7 one more time then spined for 30 seconds with the chip tab pointing in (toward the center of the mini fuge).
10. The chip was tilted at a 45-degree angle and slowly removed as much liquid as possible from the loading port by dialing the pipette. Discard the liquid.

Performing the selected the planned run

1. The browse button next to the planned run field was pressed and select the name of the plan created then pressed next.
2. The run settings were automatically populated based on the Planned Run. Confirmed that these settings were correct.
3. After enter the planned run, pressed next to verify the experimental set up pressed ok to confirm the settings.
4. After prompted by the instrument loaded and clamped the chip then pressed next.
5. At the beginning of the run, visually inspected the chip in the clamp for leaks before closing the cover.

6. After completing the calibration (~1 minute), the touch screen was indicated calibration was successful.
7. The run automatically began after 90 seconds,
8. When the run was completed, the touch screen was returned to the main menu.
